# Coronavirus infection and PARP expression dysregulate the NAD metabolome: An actionable component of innate immunity

**DOI:** 10.1074/jbc.RA120.015138

**Published:** 2021-01-13

**Authors:** Collin D. Heer, Daniel J. Sanderson, Lynden S. Voth, Yousef M.O. Alhammad, Mark S. Schmidt, Samuel A.J. Trammell, Stanley Perlman, Michael S. Cohen, Anthony R. Fehr, Charles Brenner

**Affiliations:** 1 Free Radical and Radiation Biology Program, Department of Radiation Oncology, University of Iowa, Iowa City, Iowa, USA; 2 Department of Biochemistry, University of Iowa, Iowa City, Iowa, USA; 3 Department of Chemical Physiology & Biochemistry, Oregon Health Sciences University, Portland, Oregon, USA; 4 Department of Molecular Biosciences, University of Kansas, Lawrence, Kansas, USA; 5 Department of Microbiology & Immunology, University of Iowa, Iowa City, Iowa, USA

**Keywords:** Severe acute respiratory syndrome coronavirus 2, transcriptomics, interferon, poly(ADP-ribose) polymerase, ADP-ribosylation, nicotinamide adenine dinucleotide (NAD), NAD biosynthesis, gene transcription, plus-stranded RNA virus, post-translational modification (PTM), PARP, RNA-Seq, SARS-CoV-2, COVID-19

## Abstract

Poly(ADP-ribose) polymerase (PARP) superfamily members covalently link either a single ADP-ribose (ADPR) or a chain of ADPR units to proteins using NAD as the source of ADPR. Although the well-known poly(ADP-ribosylating) (PARylating) PARPs primarily function in the DNA damage response, many noncanonical mono(ADP-ribosylating) (MARylating) PARPs are associated with cellular antiviral responses. We recently demonstrated robust up-regulation of several PARPs following infection with murine hepatitis virus (MHV), a model coronavirus. Here we show that SARS-CoV-2 infection strikingly up-regulates MARylating PARPs and induces the expression of genes encoding enzymes for salvage NAD synthesis from nicotinamide (NAM) and nicotinamide riboside (NR), while down-regulating other NAD biosynthetic pathways. We show that overexpression of PARP10 is sufficient to depress cellular NAD and that the activities of the transcriptionally induced enzymes PARP7, PARP10, PARP12 and PARP14 are limited by cellular NAD and can be enhanced by pharmacological activation of NAD synthesis. We further demonstrate that infection with MHV induces a severe attack on host cell NAD^+^ and NADP^+^. Finally, we show that NAMPT activation, NAM, and NR dramatically decrease the replication of an MHV that is sensitive to PARP activity. These data suggest that the antiviral activities of noncanonical PARP isozyme activities are limited by the availability of NAD and that nutritional and pharmacological interventions to enhance NAD levels may boost innate immunity to coronaviruses.

Disease attributed to the current novel coronavirus (CoV) outbreak (COVID-19) has rapidly spread globally, infecting more than 33 million people and killing more than a million as of late September, 2020 (1). The causative agent, severe acquired respiratory syndrome coronavirus 2, SARS-CoV-2, is transmitted largely by aerosol and liquid droplets that infect cells of the lung epithelium (2). Severe disease is thought to proceed through a combination of robust viral replication and a cytokine storm in which host inflammation damages multiple organ systems. Although many therapeutic approaches are under investigation, the evidence basis for effective prevention and treatment agents remains limited.

CoV genomes do not encode enzymes needed for ATP generation, nucleotide, amino acid, lipid, or protein synthesis and therefore depend on exploitation of host functions to synthesize and assemble virus (3, 4, 5). Cellular and viral energy generation and biosynthetic programs depend on the four NAD coenzymes, NAD^+^, NADH, NADP^+^, and NADPH, which are the central catalysts of metabolism (6). These coenzymes accept and donate electrons in essential, ubiquitous processes of fuel oxidation, lipid, nucleotide, and amino acid biosynthesis, and the generation and detoxification of reactive oxygen species. The specific roles of these coenzymes in viral replication and antiviral defenses are largely unexplored.

Several members of the PARP superfamily are interferon-stimulated genes that have been implicated in restriction of viral replication through mechanisms that are not well understood (7, 8). With the exception of the enzymatically inactive PARP13 protein, PARP isozymes have an absolute requirement for NAD^+^ (9, 10). However, rather than using NAD^+^ as an electron acceptor, PARPs use NAD^+^ as an ADPR donor in protein modification reactions (6). The canonical and best-characterized PARP isozymes, PARP1 and PARP2, form PAR largely in response to DNA damage. However, most other members of the PARP superfamily exhibit MARylation activities on target proteins (9). We previously showed that MHV infection strongly induces expression of noncanonical PARP isozymes PARP7, PARP9, PARP10, PARP11, PARP12, PARP13, and PARP14 (11). To determine whether these gene expression changes are incidental to MHV infection, facilitate MHV infection, or are part of an innate immune response against MHV, we treated cells with siRNAs to knock down expression of these genes and then analyzed the impact on MHV replication. Our data showed that PARP7 plays a role in facilitating replication. In contrast, PARP14 is required for full induction of IFN-β expression (11, 12), suggesting that PARP14 is directly involved in establishing the innate immune response in CoV-infected cells.

Most CoV genomes encode 16 nonstructural proteins (nsps) (3, 4, 5). Nsp3 contains a macrodomain, herein termed the CoV ADPR hydrolase (CARH) that removes ADPR modifications from acidic amino acids on protein targets. Thus, CARH reverses the modification that is installed by the IFN-induced activities of MARylating PARP isozymes (13). CARH activity is required for virulence *in vivo* using mouse models of both MHV and SARS-CoV (13, 14, 15). Moreover, an active site mutation that ablates CARH activity in MHV resulted in a virus that replicates poorly in primary bone marrow–derived macrophages (BMDMs) (11). We further identified PARP12 and PARP14 as CoV-induced interferon-stimulated genes that are required for depressed replication of CARH mutant viruses, indicating that their activity is opposed by CARH-mediated reversal of MARylation (11).

In support of the antiviral roles of MARylating PARP isozymes, PARP12 was shown to promote degradation of nsp1 and nsp3 in Zika virus infection (16). PARP12 has also been shown to inhibit a wide variety of RNA viruses, including several alphaviruses, which also contain nsp3-encoded ADPR hydrolase activities (17, 18). These observations suggest that key events in the innate immune response to viral infections are played out in the infected cell's NAD metabolome.

To determine whether the dramatic up-regulation of PARPs seen following MHV infection is conserved among CoVs and also whether cellular infection by CoVs disturbs NAD homeostasis, we analyzed transcriptomic data from SARS-CoV-2–infected human cell lines and organoids, SARS-CoV-2–infected ferrets, a lung biopsy from a person who died of COVID-19, and bronchoalveolar lavage fluid (BALF) from healthy individuals as well as those infected with COVID-19. These data indicate that the same noncanonical PARPs induced by MHV are induced by SARS-CoV-2 and that *in vivo* infection with SARS-CoV-2 down-regulates synthesis of NAD from tryptophan and nicotinic acid (NA) while up-regulating synthesis capacity from NAM and NR. We further show disturbances to the NAD transcriptome scale with viral load. Although noncanonical PARP isozymes are known to use NAD^+^ to MARylate target proteins, it had not been reported that expression of these enzymes can drive down cellular NAD^+^. Here we show that PARP10 overexpression is sufficient to depress cellular NAD^+^ and that the MARylating activities of PARP10, PARP12, and PARP14 can be pharmacologically increased by enhancing NAD salvage synthesis with SBI-797812 (SBI), a NAMPT activator (19).

Although the essentiality of CARH for viral pathogenesis argues for cellular NAD and noncanonical PARP induction as antiviral, it remained conceivable that a depressed cellular NAD metabolome is an adaptive antiviral response to restrict viral biosynthetic processes. We therefore established a cellular system to test whether increased NAD status opposes MHV infection. Consistent with our transcriptomic analysis that CoV infection down-regulates NA salvage and up-regulates NAM and NR salvage, we found, using an MHV mutant virus sensitive to PARP activity, that NA minimally inhibited viral replication, whereas NAM, SBI, and a clinically tested preparation of NR (Niagen) (20, 21, 22) strongly inhibit MHV replication. The data justify further analysis of how nutritional and therapeutic modulation of NAD status may potentially restrict viral infection by boosting PARP activity.

## Results

### SARS-CoV-2 infection of human lung systems induces a noncanonical PARP isozyme transcriptional program

**Figure 1:**
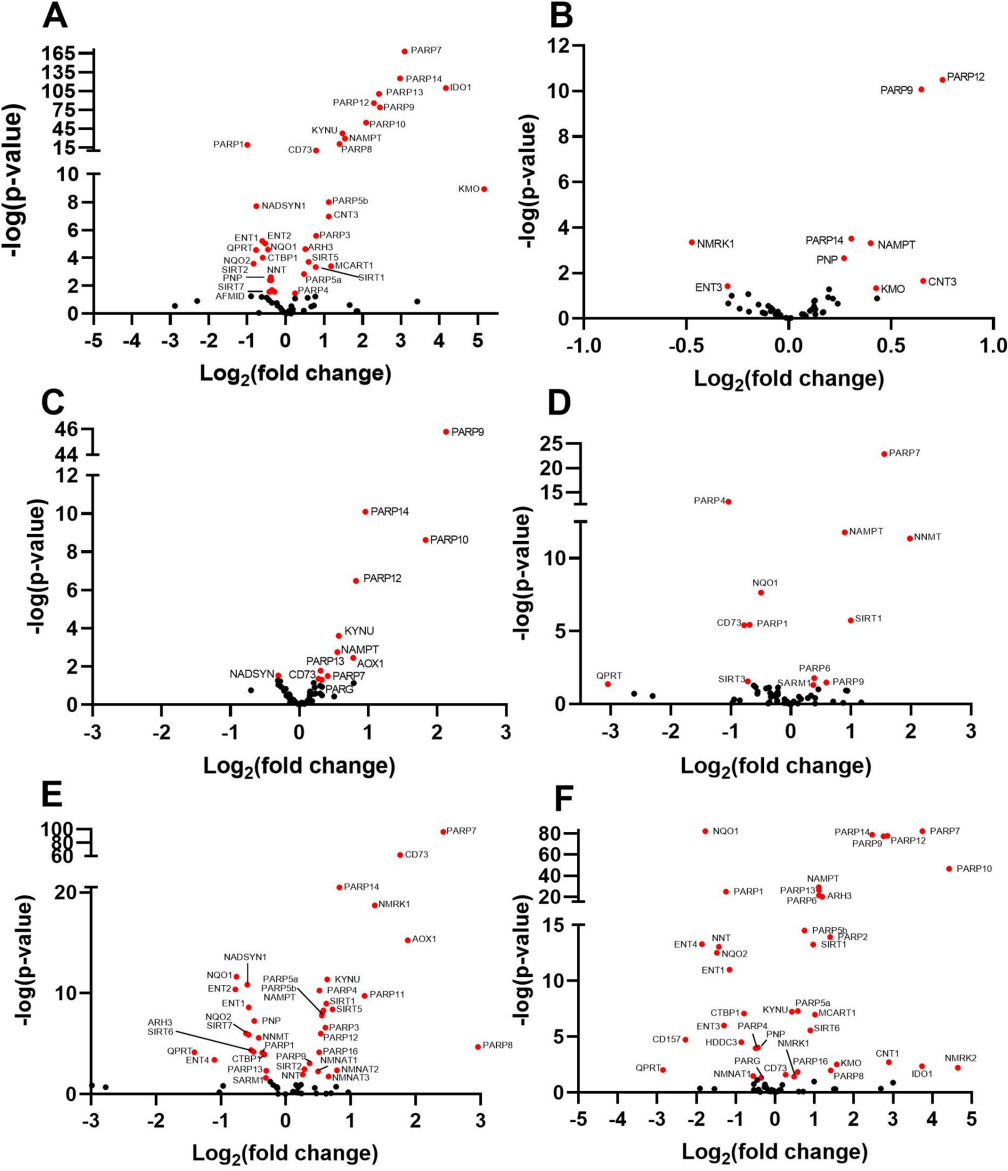
**SARS-CoV-2 dysregulates the NAD gene set *in vitro* as a function of viral load.** Differential expression analysis was performed on RNA-Seq data with respect to a 71 gene set representing the NAD transcriptome (Table S1). *A*–*F*, depicted are volcano plots representing normalized relative expression *versus* −log(P) with respect to mock infected in (*A*) human Calu3 lung cancer cells (MOI = 2), (*B*) NHBE cells (MOI = 2), (*C* and *D*), A549 cells at low MOI = 0.2 without and with introduction of ACE2 expression, respectively, and (*E* and *F*) A549 cells at high MOI = 2 without and with introduction of ACE2 expression, respectively. Further information is available in supporting material 2–7.

MHV infection in murine BMDMs launches a transcriptional program that induces transcription of noncanonical PARP isozymes PARP7, PARP9, PARP10, PARP11, PARP12, PARP13, and PARP14 by >5-fold (11, 12). To determine whether SARS-CoV-2 also dysregulates the NAD system upon infection, we assembled and analyzed a set of 71 genes that encode the enzymes responsible for conversion of tryptophan, NA, NAM, and NR to NAD^+^, plus the enzymes responsible for NAD(H) phosphorylation, NADP(H) dephosphorylation, NAD^+^-dependent deacylation, MARylation, PARylation, cADP-ribose formation, nicotinamide methylation/oxidation, and other related functions in transport, binding, redox, and regulation (supporting material 1). We then analyzed RNA-Seq data from SARS-CoV-2 infection of three human lung cell lines, Calu-3, normal human bronchial epithelia (NHBE), and A549 (Fig. 1, *A***–***C*) (23). Calu-3 cells exhibit the most robust response, inducing PARP7, PARP9, PARP10, PARP12, and PARP14 more than 4-fold as well as PARP3, PARP4, PARP5a, PARP5b, and PARP8 to a lesser but statistically significant degree (Fig. 1*A*). In NHBE cells, SARS-CoV-2 induces transcription of PARP9, PARP12, and PARP14; in A549 cells it induces PARP9, PARP10, PARP12, and PARP14, with lesser effects on PARP7 and PARP13 (Fig. 1, *B* and *C*).

### Transcriptional dysregulation of NAD metabolism scales with viral titer

Angiotensin-converting enzyme 2 (ACE2) serves as a receptor for SARS-CoV-2 entry (24). To determine the effect of overexpression of ACE2 or greater viral exposure on the NAD transcriptome, we compared high quality RNA-Seq data from control or ACE2-overexpressing A549 cells infected at low (0.2) or high (2) multiplicity of infection (MOI). Greater viral exposure dysregulated more NAD-related genes (Fig. 1, *D*–*F*). The viral load–dependent changes came in three types. First, PARP7 is strongly dependent on viral load, being minimally induced in A549 cells infected at a low MOI (Fig. 1*C*). However, in cells expressing ACE2 or infected at high MOI, PARP7 was one of the most highly induced PARP isozymes (Fig. 1, *D***–***F*). Second, as ACE2 or more virus was added, transcription of more PARP isozymes was disturbed. ACE2 and higher MOI both down-regulated expression of PARP1 while they up-regulated expression of 12 of the other 16 PARP superfamily members (Fig. 1*F*). Third, on the basis of transcriptional changes, viral infection alters expression of NAD biosynthesis pathways. ACE2 and higher MOI both down-regulated expression of QPRT, which is required for the conversion of quinolinate to nicotinic acid mononucleotide in the *de novo* biosynthetic pathway that originates with tryptophan. Similarly, NADSYN, which is required for synthesis of NAD from both tryptophan and NA, is down-regulated by viral infection, suggesting that CoV infection might disadvantage repletion of the NAD metabolome from either tryptophan or NA. In contrast, NAMPT, which is required for NAD synthesis from NAM, was consistently up-regulated in SARS-CoV-2–infected cells, and NMRK1, which is required for NAD synthesis from NR, was up-regulated when cells were infected at high MOI. In addition, the normally minimally expressed NR kinase gene, NMRK2, was up-regulated in the ACE2-overexpressing cells infected at high MOI.

### Primary 3D cell culture and in vivo SARS-CoV-2 infection disturb noncanonical PARP isozyme expression and NAD biosynthesis

**Figure 2:**
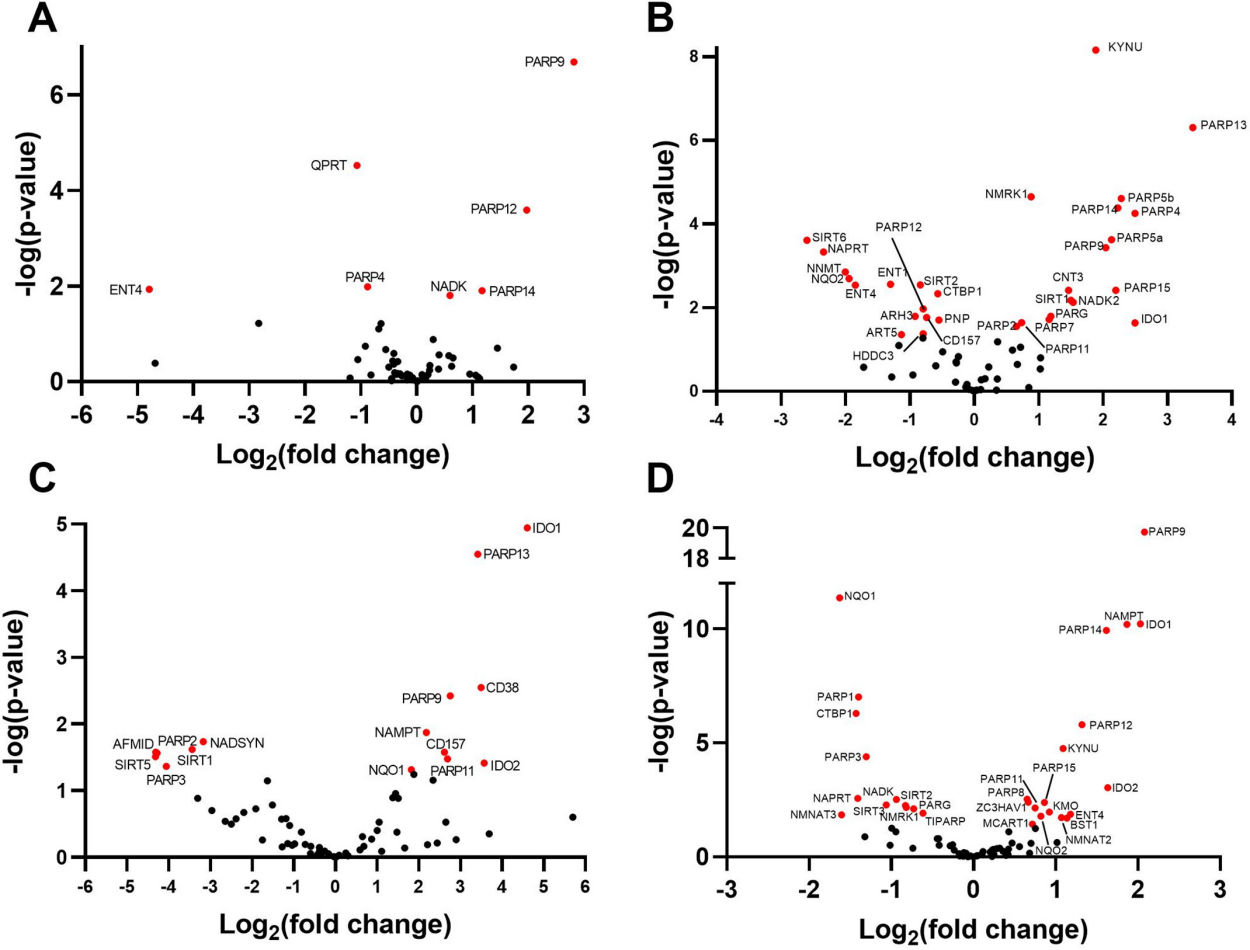
**SARS-CoV-2 dysregulates the NAD gene set *in vivo.****A*–*D*, differential expression analysis was performed on RNA-Seq data with respect to mock infected in (*A*) expanding enterocytes (MOI = 1), (*B*) ferret trachea infected with SARS-CoV-2, (*C*) lung of a diseased COVID-19 patient *versus* a control lung sample, and (*D*) BALF from SARS-CoV-2 infected *versus* healthy control human patients. Further information is available in supporting material 8–11.

Based on the finding that SARS-CoV-2 infects gut enterocytes, we analyzed RNA-Seq data obtained from infected enterocyte organoids (25). Similar to the lung cell lines and to the transcriptional effect of MHV infection, enterocyte organoids infected with SARS-CoV-2 induce PARP9, PARP12, and PARP14 (Fig. 2*A*).

Ferrets have been shown to be permissive to SARS-CoV-2 infection (26) and are being used as a system to probe host responses as well as potential preventative and therapeutic agents. To determine whether PARP up-regulation following SARS-CoV-2 infection is also observed *in vivo*, we probed high-quality RNA-Seq data from the tracheas of mock and 3-day SARS-CoV-2–infected ferrets (23) and found that the noncanonical PARP induction program is conserved in this relevant animal model (Fig. 2*B*). Specifically, PARP4, PARP5, PARP9, PARP13, PARP14, and PARP15 were all >4-fold induced with significant but lesser induction of PARP7 and PARP11. In ferret tracheas, we observed other significant alterations to the NAD gene set. The data indicate that transcription of NMRK1 and concentrative nucleoside transporter CNT3 are induced in response to CoV infection, suggesting increased capacity for uptake and conversion of NR to NAD^+^ and NADP^+^ (27). Notably, in mouse models of damaged brain and heart, up-regulation of NMRK gene expression is associated with therapeutic efficacy of NR (28, 29). Additionally, the ferret data show strongly depressed NNMT expression; by decreasing NAM methylation, this gene expression change could promote NAM salvage (30) and the efficiency of NAD-boosting by NR or NAMPT activators (20), representing a homeostatic attempt of virally infected cells to maintain their NAD metabolome (31).

### COVID-19 patient samples recapitulate the PARP induction program seen in ferrets and in vitro

Finally, although ferrets are susceptible to infection by SARS-CoV-2, they do not progress to the serious disease seen in humans (26). We therefore examined the NAD gene set in RNA-Seq data from the lung of an individual who died of COVID-19 (23). Although lacking the replicates and the synchrony of the ferret RNA-Seq data, the human data showed that PARP9, PARP11, and PARP13 were up-regulated in the deceased individual's lung (Fig. 2*C*).

Finally, we analyzed RNA-Seq data obtained from the BALF of 430 SARS-CoV-2–infected people and 54 controls (32). Consistent with previous findings, PARP9 was up-regulated nearly 4-fold whereas PARP12 and PARP14 were up-regulated more that 2-fold (Fig. 2*D*).

Notably, NAD biosynthetic gene changes were conserved *in vivo* with NAMPT and NMRK1 gene expression increased by viral infection in ferrets, and NAMPT increased in the human patient samples. Further data on transcriptomic alterations to NAD synthesis in all nine data sets are provided in supporting material 12.

### PARP10 overexpression is sufficient to depress NAD^+^ levels

**Figure 3:**
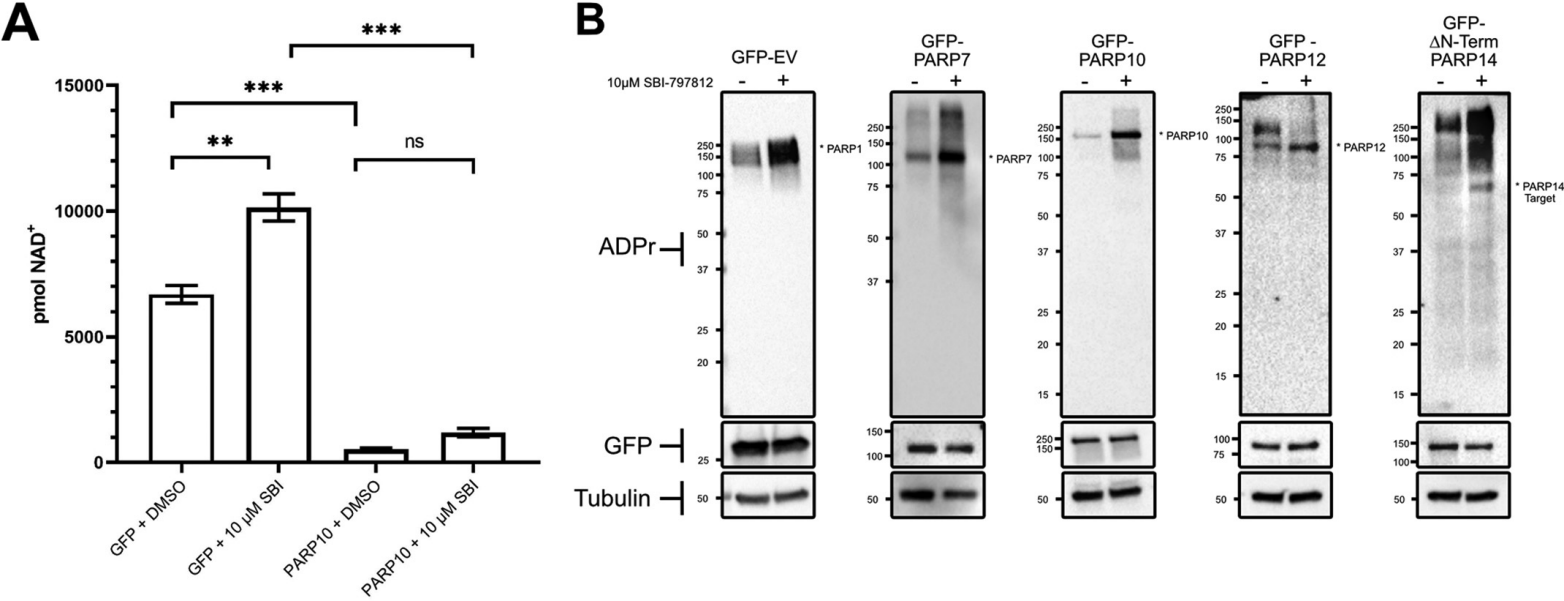
**PARP10 overexpression is sufficient to depress cellular NAD^+^ levels whereas SBI enhances activities of overexpressed PARP7, PARP10, PARP12, and PARP14.***A*, HEK 293T cells were grown with the indicated expression plasmids for GFP or PARP10 and treated with NAMPT activator (10 μm SBI). *n* = 3 for each group. *Error bars* represent S.E., *p*-values are from an unpaired two-tailed *t* test. See also supporting information 13. *B*, GFP, PARP7, PARP10, PARP12, and PARP14-expressing HEK293 cells were treated with 10 μm SBI and cells were collected 18 h later. Western blotting using indicated antibodies indicates that SBI promotes PARP7, PARP10, PARP12, and PARP14 activity. *n* = 3. Representative blots of three independent experiments are shown. **, *p*≤ 0.01; ***, *p*≤ 0.001.

It is well known that PARP1 activation by DNA damage greatly increases its catalytic activity, leading to depression of cellular NAD^+^ and ATP (9, 10). It is less clear whether transcriptional induction of the MARylating enzymes such as PARP10 that are induced substantially by viruses including SARS-CoV-2 might disturb cellular NAD^+^. To test whether overexpression of a MARylating enzyme is sufficient to disturb the NAD metabolome, we performed targeted quantitative NAD analysis with LC–MS/MS using internal ^13^C standards (33) on HEK 293T cells expressing either GFP or a GFP-PARP10 fusion. We found that overexpression of GFP-PARP10 significantly depressed NAD^+^ compared with overexpression of GFP alone (Fig. 3*A*). We next determined if the PARP10-mediated loss in NAD^+^ could be restored by increasing cytosolic NAD^+^ synthesis with SBI, a small molecule activator of NAMPT, which promotes NAM salvage (19). SBI increased steady-state levels of NAD^+^ in GFP-expressing cells but did not significantly boost NAD^+^ in PARP10-expressing cells, indicating that PARP10 expression is sufficient to limit cellular NAD^+^ (Fig. 3*A*).

### Enhanced NAD salvage increases the activity of PARP isozymes induced by MHV & SARS-CoV-2 infection

The ability of SBI to elevate cellular NAD^+^ in GFP-expressing cells but not in PARP10-expressing cells suggested that overexpressed PARP isozymes are active at lower levels of cellular NAD^+^ but left open the question of whether overexpressed PARP isozymes have enzymatic activities that are limited by the depressed NAD^+^ status that they confer. To determine whether the activity of PARP7, PARP10, PARP12, and PARP14 can be increased by NAMPT stimulation, we measured ADPR-modified protein levels by Western blotting in HEK 293T cells overexpressing GFP, or GFP-tagged PARP7, PARP10, PARP12, or PARP14 in the presence or absence of SBI. Consistent with the ability of SBI treatment to elevate cellular NAD^+^, SBI enhanced PARP1 autoPARylating activity in GFP-expressing cells. SBI treatment resulted in a striking increase in PARP10 activity as evidenced by enhanced PARP10 autoMARylation. A similar result was observed with PARP7 and PARP12 albeit to a lesser degree. Finally, SBI treatment of PARP14-expressing cells resulted in a significant increase in PARP14 target MARylation (autoMARylation was not detected under these conditions) (Fig. 3*B*). These results indicate that activities of overexpressed PARP isozymes are limited by cellular NAD^+^ levels and can be enhanced pharmacologically.

### MHV infection drives down cellular NAD^+^ and NADP^+^ in infected cells

**Figure 4:**
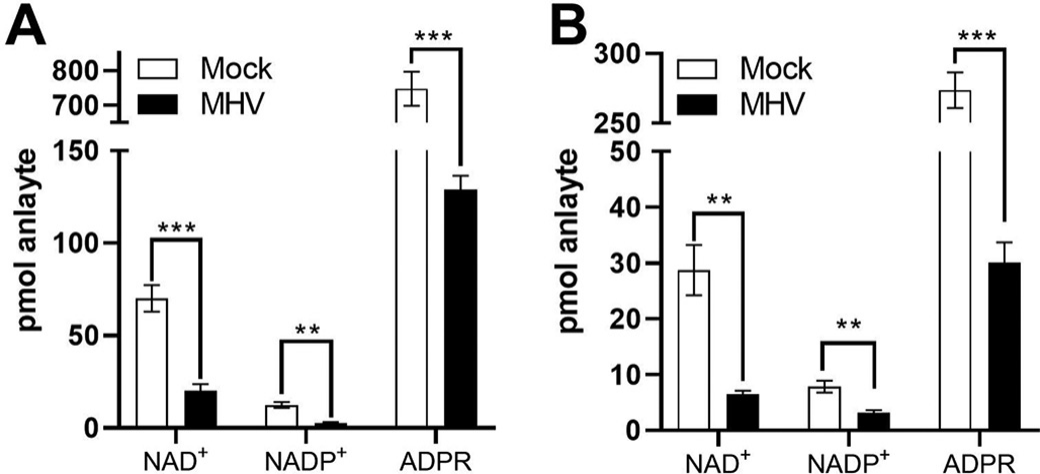
**MHV infection disturbs the NAD metabolome.***A* and *B*, DBT cells (*A*) and BMDM cells (*B*) were mock infected or infected with MHV at a MOI of 3 pfu/cell and cells were collected at 12 h post infection. *n* = 3–4 mock; *n* = 4 MHV. *Error bars* represent S.E., *p*-values are from unpaired two-tailed *t* test. **, *p* ≤ 0.01; ***, *p* ≤0.001. See also supporting material 14 and 15.

Given that CoVs consistently induce members of the PARP superfamily at the mRNA level, we asked whether MHV infection alters the NAD metabolome. MHV is a model CoV that can be propagated under BSL-2 conditions, allowing us to obtain samples needed for quantitative analysis of the NAD metabolome. We infected delayed brain tumor (DBT) cells with MHV-A59 at an MOI of 3 and subjected the cells to quantitative targeted NAD metabolomics 12 h after infection (33). Infection led to a >3-fold depression of cellular NAD^+^ and NADP^+^ with respect to control after a mock infection (Fig. 4*A*). To address the possibility that these effects are specific to a cancer cell line and not a primary viral target cell population, we prepared BMDM from C57BL/6 mice and infected them with MHV-A59 at an MOI of 3. Similar to DBT cells, we observed a greater than 3-fold depression of cellular NAD^+^ and NADP^+^ with respect to control cells at 12 h (Fig. 4*B*). Thus, in primary cells that represent an authentic CoV target cell population, MHV depresses levels of NAD coenzymes.

### NAD boosting compounds decrease replication of a CARH-mutant MHV

We previously reported that the CARH domain of MHV and SARS-CoV Nsp3 proteins is required for maximum replication and pathogenesis *in vivo* (13, 14, 15). Moreover, the N1347A active site mutation that ablates the ADPR hydrolase activity of CARH and *in vivo* infectivity of MHV also resulted in a virus that replicates poorly in primary BMDM cells but reaches similar peak titers as WT virus in a transformed 17Cl-1 fibroblast cell line (11, 14). These data suggest that a CARH inhibitor would be completely antiviral *in vivo* and would show some activity in dampening replication in cellular infection models.

Our data established that noncanonical PARPs are consistently induced by CoV infection (Figure 1, Figure 2), can depress cellular NAD^+^ and have their MARylation activities limited by cellular NAD^+^ (Fig. 3), and have known antiviral activities (16, 17, 18). These data suggest that boosting cellular NAD through the transcriptionally up-regulated NAMPT and NMRK pathways would have antiviral activity. However, it remained a possibility that the antiviral response of infected cells evolved to drive down cellular NAD^+^ and NADP^+^ to rob invading viruses of biosynthetic capacity.

**Figure 5:**
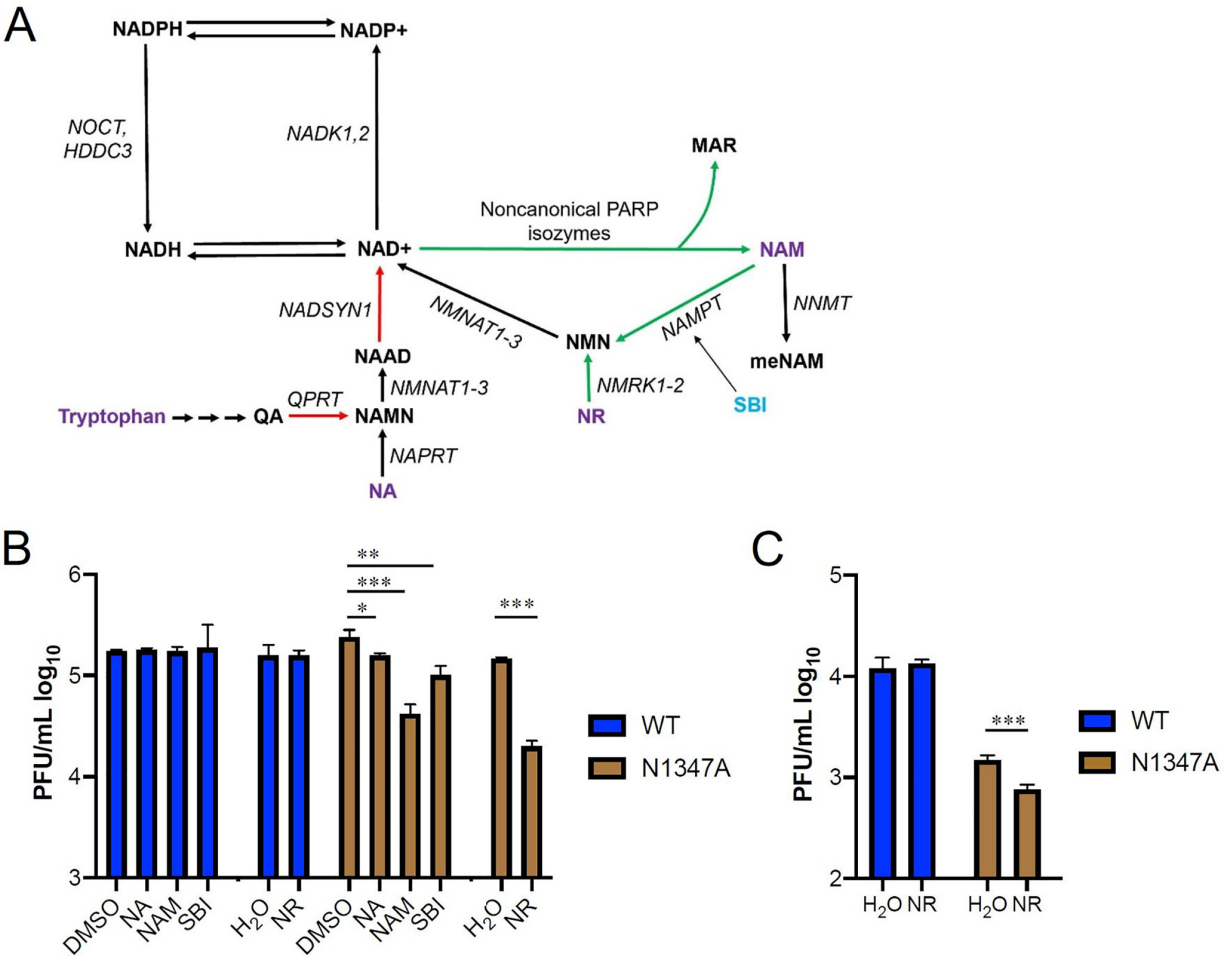
**Boosting NAD^+^ levels depresses replication of CARH mutant MHV.***A*, NAD biosynthetic pathways. *Red arrows* depict gene expression that is depressed by SARS-CoV-2. *Green arrows* depict gene expression that is increased by SARS-CoV-2. *B*, 17Cl-1 cells were infected with 0.1 pfu/cell WT or N1347A MHV and either mock treated (DMSO or H_2_O) or treated with NA, NAM, SBI, or NR as described in “Experimental procedures.” DMSO served as a solvent control for NAM, SBI, and NA, whereas H_2_O served as a solvent control for NR. Cells were collected at 18 hours post-infection (*hpi*) and analyzed for virus replication by plaque assay. Data are representative of two independent experiments, *n* = 3 biological replicates. *C*, BMDMs were infected with 0.1 pfu/cell WT or N1347A MHV and treated with H_2_O or treated with NR as described in “Experimental procedures.” Cells were collected at 18 hpi and analyzed for virus replication by plaque assay. Data are representative of two independent experiments. *n* = 4 biological replicates. *, *p* ≤ 0.05; **, *p* ≤ 0.01; ***, *p* ≤ 0.001.

To test the hypothesis that NAD-boosting interventions would depress viral replication in sensitive cellular assays, we measured MHV N1347A infection of BMDM and 17Cl-1 systems as a function of addition of NA, NAM, SBI, or NR (Niagen) (Fig. 5*A*). As previously reported, N1347A reached similar peak titers as WT virus in 17Cl-1 cells. However, the addition of NA, NAM, SBI, and NR all significantly decreased its replication (Fig. 5*B*). Consistent with increased NAMPT and NMRK gene expression and depressed NADSYN expression in SARS-CoV-2–infected cells (Figure 1, Figure 2), NAM, SBI, and NR had significantly greater effects on N1347A replication than NA. NAM, SBI, and NR decreased N1347A replication by 8.3-, 4.4-, and 6.4-fold, respectively, whereas NA decreased its replication by only 1.9-fold (Fig. 5*B* and Table 1). Consistent with the permissivity of 17Cl-1 cells to infection (11, 14), these treatments did not affect WT virus replication in 17Cl-1 cells.

**Table 1:** Impact of NAD-boosting compounds on N1347A replication The average -fold reductions in viral titer of N1347A in two independent experiments on 17Cl-1 or BMDM cells. The *p*-values are from an unpaired two-tailed *t*-test of data from both experiments combined

Compound	Fold reduction in N1347A viral titer	*p* value
NA	1.9	4.4E-2
SBI	4.4	1.7E-3
NAM	8.3	6.3E-4
NR	6.4	8E-5
NR (BMDM)	2.7	<1E-6

Next we tested the ability of NR to reduce N1347A replication in BMDM cells. Here, N1347A with no treatments had a replication defect of 4.8-fold compared with WT virus, similar to our previous report (11). Consistent with the view that higher NAD^+^ status depresses viral infectivity in cellular assays, addition of NR to cultures infected by N1347A further depressed replication by 2.7-fold (Fig. 5*C* and Table 1).

Our data indicate that (i) CoV infection initiates the expression of multiple noncanonical PARP isozymes and dysregulates other genes involved in NAD metabolism, (ii) CoV infection and PARP10 expression can dramatically decrease NAD^+^ accumulation, and (iii) NAD^+^ boosting agents can improve PARP isozyme function and decrease the replication of a CoV that is sensitive to MARylating activities.

## Discussion

SARS-CoV-2 is a highly infectious agent that constitutes a severe threat to public health (2). Morbidity and mortality data make it clear that age, smoking status, and multiple preexisting conditions greatly increase the frequency of serious illness and death (34). There is an abundance of data from model systems and humans that age and conditions of metabolic stress including obesity and type 2 diabetes (35), smoking (36), heart failure (29), nerve damage (37), and central brain injury (28) challenge the NAD system in affected tissues. Although PARP1 was known to be a significant consumer of NAD^+^, we showed that noncanonical PARP isozymes are consistently up-regulated by CoV infections, that PARP10 overexpression can depress the NAD metabolome, and that four noncanonical PARP isozymes that are induced by SARS-CoV-2 have MARylating activities that are limited by cellular NAD status. In addition, the NAD metabolome was depressed by MHV infection. Although the degree of depression of the NAD metabolome is surely sensitive to time and MOI, the >3-fold depression seen in this study is precedented in HIV and *herpesvirus* infections (38, 39).

Although the genetic requirement for CARH for viral replication *in vivo* (11, 13, 14, 15) strongly suggested that higher NAD^+^ status would be protective, it was also conceivable that PARP-mediated cellular repression of the NAD metabolome constituted a mechanism for cells to deprive the virus of anabolic capacity. However, here we showed that boosting NAD^+^ through the NR and NAMPT pathways depresses replication in a CARH-mutant cellular infection model with no effect on replication of WT virus. Further experiments will be required to identify the key MARylated targets that are potentially enhanced by NAD-boosting and reversed by CARH activities.

Based on gene expression data in response to SARS-CoV-2 infection (Figure 1, Figure 2), NAD boosting approaches involving increased *de novo* or NA-dependent synthesis are unlikely to be strongly effective because they require expression of genes such as QPRT, NADSYN, and NAPRT (40) that are depressed by SARS-CoV-2 infection. Consistent with gene expression changes, in the cellular infection system, we showed that addition of NA only modestly reduced virus replication (Fig. 5, *B* and *C* and Table 1).

Based on gene expression data as well as MHV cellular infection data, NAM, NAMPT activators, and NR have similar potential to be strongly protective *in vivo*. However, there are a few caveats. First, that at pharmacological doses, NAM has the potential to function as a PARP inhibitor (41). Second, NAMPT is considered a driver of pulmonary vascular remodeling and potentially a target to be inhibited to maintain lung health of some people at risk for COVID-19 (42). Thus, to maximize the likelihood of success in human CoV prevention and treatment trials, care should be taken to carefully compare efficacy and dose-dependence of NR, SBI, and NAM with respect to control of cytokine storm and antiviral activities *in vivo*.

The cellular results presented herein warrant the testing of NAD boosting agents in the context of *in vivo* CoV infections. In addition to animal trials, the safety of various forms of vitamin B3 should allow rapid clinical assessments of NAD boosters to be evaluated in two placebo-controlled contexts.

First, we suggest that improved NAD status could help blunt the severity of infection by sustaining PARP-dependent IFN signaling in the face of the self-limiting nature of cellular NAD during infection and by limiting the storm of inflammatory cytokines that is typically associated with serious disease (43). In a small placebo-controlled clinical trial designed to address the oral safety and activity of Niagen NR in older men, it was discovered that 1 g of NR per day depresses levels of IL-6, IL-5, and IL-2 (22). Based on these findings, we suggest that NAD boosters be tested on hospitalized and nonhospitalized COVID-19 patients with primary end points of disease recovery, oxygenation, and cytokine levels. Similar approaches might also be tested in the context of other viral infections that induce noncanonical PARP isozymes and/or encode viral ADP-ribosylhydrolase activities (17, 18).

Second, as there is no standard of care for housemates or caretakers of infected people, we suggest that NAD-boosting could be tested as a placebo-controlled intervention for people in the proximity of quarantined or hospitalized COVID-19 individuals; the primary end point would be protection against infection.

Although caution should be exercised with respect to any preventative measure, NAD boosting approaches have the potential to support the innate immune system and address the age, smoking, and comorbid conditions associated with worse SARS-CoV-2 outcomes (34). The potential societal benefit of a safe and readily available molecule to support prevention and public health is hard to overstate, especially as new outbreaks of COVID-19 emerge.

## Experimental procedures

### RNA-Seq analysis

Sequence counts for Fig. 1, including cell lines infected with SARS-CoV-2 (strain USA-WA1/2020), were derived from published RNA-Seq (GSE147507) (23). Briefly, A549 (± ACE2), Calu3, and NHBE cell lines were infected with either Mock or SARS-CoV-2 virus for 24 h. Data for Fig. 2 were drawn from several sources. First, expanding enterocyte data (Fig. 2*A*) were obtained from supplementary Table 2 from Lamers *et al.* (25). Briefly, organoids were infected with SARS-CoV-2 at an MOI of 1 in expansion medium for 60 h. RNA was collected, and differential gene expression analysis was performed using the DESeq2 package. The ferret (Fig. 2*B*) and deceased human data (Fig. 2*C*) were also obtained from GSE147507. Ferrets were infected with SARS-CoV-2 at a pfu of 5 × 10^4^. More information regarding sample preparation and data processing can be found at GEO accession GSE147507. Finally, the human BALF data Fig. 2*D* were obtained from GSE152075. Briefly, samples were obtained from nasopharyngeal swabs of from 430 male and female individuals with SARS-CoV-2 infection and 54 controls (32). NHBE (Fig. 1*B*) and A549 (low MOI) (Fig. 1*C*) data were gathered from supplementary Tables 2 and 1 from Blanco-Melo *et al.* (23). Genes with status = low or outlier were not considered for analysis. All other datasets were analyzed from GEO data using DESeq2 by the University of Iowa Bioinformatics Core (see “Data and code availability”). Genes with a *p*-value of zero were treated as having a *p*-value equal to the next lowest *p*-value in that dataset. Genes with *p* < 0.05 (−log(p) > 1.30) were considered statistically significant. Graphs were generated using GraphPad Prism version 8.

### Cell culture

DBT, 17Cl-1, HEK293T, and HeLa cells expressing the MHV receptor carcinoembryonic antigen-related cell adhesion molecule 1 (a gift from Dr. Thomas Gallagher, Loyola University, Chicago, IL) were grown in Dulbecco's modified Eagle's medium (DMEM) supplemented with 10% FBS, HEPES, sodium pyruvate, nonessential amino acids, l-glutamine, penicillin, and streptomycin. To create BMDMs, bone marrow cells were harvested from C57BL/6 mice and differentiated by incubating cells with 10% L929 cell supernatants and 10% FBS in RPMI media for 7 days. Cells were washed and replaced with fresh media every day after the 4th day. For analysis of the NAD metabolome HEK 293T cells were transfected with 1 μg of pEGFP-C1 Empty Vector or pEGFP-C1-CMV-PARP10 using CalPhos Mammalian Transfection Kit (Takara Bio). 6 h later the cells were treated with chemical treatments in DMEM + 10% FBS at 37°C 5% CO_2_ for 18 h.

### Mice

Animal studies were approved by the University of Kansas Institutional Animal Care and Use Committee (IACUC) as directed by the Guide for the Care and Use of Laboratory Animals (Protocol no. 252-01). Anesthesia or euthanasia were accomplished using ketamine/xylazine. Pathogen-free C57BL/6 mice were purchased from The Jackson Laboratory and maintained in the animal care facility at the University of Kansas.

### Virus infection

Recombinant WT (rJIA-GFP*rev*N1347) and N1347A (rJ-IA-GFP-N1347A) MHV were previously described (3). Both viruses expressed eGFP. MHV-A59 was described previously (44). All viruses were propagated on 17Cl-1 as described previously (11). DBT, 17Cl-1, and BMDM cells were infected as described in the figure legends with a 1-h adsorption period, before virus was removed from the well and replaced with fresh media. For NAD analysis, DBT cells were washed with PBS and replenished with serum-free DMEM prior to infection with no supplements except penicillin and streptomycin and were maintained in serum-free media throughout the infection. For treatments with NAD modulating compounds, 10 μm NA, NAM, and SBI-797812 were added immediately following the adsorption phase. For NR experiments, 100 nmol of NR was added to cells in 24-well plates in serum-free media 4 h prior to infection, removed during the adsorption phase, then another 100 nmol NR in serum-free media was added following the adsorption phase. Another 100 nmol of NR was then added directly to the media again at 12 hpi. Cells and supernatants were collected at 18 hpi and viral titers were determined by plaque assay.

### Quantitative NAD metabolomics

NAD metabolites were quantified against internal standards in two LC–MS/MS runs as described in Ref. 33.

### Western blot analysis of PARP MARylation

HEK 293T were transfected with 3 μg pEGFP-C1 empty vector, pEGFP-C1-CMV-PARP7, pEGFP-C1-CMV-PARP10, pEGFP-C1-CMV-PARP12, or pEF1-EGFP-C1-PARP14(553–1801) via CalPhos Mammalian Transfection Kit. 6 h later the cells were treated with chemical treatments in DMEM + 10% FBS at 37°C 5% CO_2_ for 18 h. Cells were washed in PBS and lysed in 50 mm HEPES, pH 7.4, 150 mm NaCl, 1 mm MgCl_2_, 1 mm TCEP, 1% Triton X-100 with the addition of Protease Inhibitors (Roche), 30 μm rucaparib (Selleck), and 1 μm PDD0017273 (Sigma). Lysates were microcentrifuged for 15 min at 4°C and quantified by Bradford assay, and supernatants were transferred to a new tube with 4× SDS sample loading buffer (0.2M Tris-HCl, pH 6.5, 4% BME, 8% w/v SDS, 0.0.8% Bromphenol Blue, 40% glycerol). Samples were resolved via SDS-PAGE and transferred to nitrocellulose. Blots were blocked with 5% milk-PBST for 30 min, incubated O/N in primary antibody (rabbit pan-ADPr, 1:1000, Cell Signaling Technology E6F6A; rabbit GFP, 1:1000, Chromotek PABG1-100; mouse tubulin, 1:1000; Cell Signaling Technology DM1A). Primary incubation was followed with HRP-conjugated secondary antibodies (rabbit-HRP 1:10000, The Jackson Laboratory 111-035-144; mouse-HRP, 1:5000, Invitrogen 62-6520). Blots were developed by chemiluminescence and imaged on a ChemiDoc MP system (Bio-Rad). Blot analysis was performed in Image-Laboratory (Bio-Rad).

### PARP plasmids

pEGFP-C1-CMV-PARP7, pEGFP-C1-CMV-PARP10, pEGFP-C1-CMV-PARP12, and pEF1-EGFP-C1-PARP14[553-1801] were generated with standard restriction digest cloning.

## Data and code availability

Transcriptomic data for all NAD-related genes are provided in supporting material 2–11. Primary RNA-Seq data are from published studies (23, 25, 32). NAD metabolomics data are provided in supporting material 13 and 14. Code for the generation of data in supporting material 2, 5–7, and 9–11 are provided.
