# Supplementary material for: Coronavirus infection and PARP expression dysregulate the NAD metabolome: An actionable component of innate immunity
Source: J Biol Chem. 2021 Jan 13;295(52):17986–96. doi: 10.1074/jbc.RA120.015138 (PMC7834058; doi:10.1074/jbc.RA120.015138)
Supplement: Supplementary file 1 [file mmc1.zip › 161984_1_supp_605516_qh554s.html]

# Brenner RNA-seq Covid 19 Series 7 analysis

#### Henry Keen

#### 04.20.2020

# libraries

```
library(dplyr)
library(stringr)
library(DESeq2)
library(ggplot2)
library(ggrepel)
library(tibble)
library(knitr)
library(kableExtra)
library(grid)
library(gridExtra)
```

## input parameters

```
my_series  <- "Series7"

my_control <- "Mock"
my_exp   <- "SARS.CoV.2"
```

# Import data

```
## get counts data

file <- "./04_rawcounts_2020_04_20_modified/GSE147507_RawReadCounts_Human.tsv"

all(file.exists(file))
```

```
## [1] TRUE
```

```
cts <- as.matrix(read.csv(file,sep="\t",row.names="Gene"))


## get metadata

coldata_file <- "./05_coldata_2020_04_20/GSE147507.human.coldata.txt"

coldata <- read.table(coldata_file, sep='\t', header=TRUE)

rownames(coldata) <- coldata$sample


## check
rownames(coldata) %>% length()
```

```
## [1] 78
```

```
colnames(cts) %>% length()
```

```
## [1] 78
```

```
all(rownames(coldata) == colnames(cts))
```

```
## [1] TRUE
```

```
## filter metadata to human data

coldata_original <- coldata

coldata <- coldata %>% filter(series ==  my_series) 

rownames(coldata) <-coldata$sample

## filter counts
cts <- cts[, as.vector(coldata$sample)]

## recheck
cts %>% head()
```

```
##           Series7_Calu3_Mock_1 Series7_Calu3_Mock_2 Series7_Calu3_Mock_3
## DDX11L1                      0                    0                    0
## WASH7P                      25                   60                   84
## FAM138A                      0                    0                    0
## FAM138F                      0                    0                    0
## OR4F5                        0                    0                    0
## LOC729737                   65                  184                  435
##           Series7_Calu3_SARS.CoV.2_1 Series7_Calu3_SARS.CoV.2_2
## DDX11L1                            1                          0
## WASH7P                            47                         32
## FAM138A                            0                          0
## FAM138F                            0                          0
## OR4F5                              0                          0
## LOC729737                        271                        137
##           Series7_Calu3_SARS.CoV.2_3
## DDX11L1                            0
## WASH7P                            41
## FAM138A                            0
## FAM138F                            0
## OR4F5                              0
## LOC729737                        265
```

```
coldata %>% head()
```

```
rownames(coldata) %>% length()
```

```
## [1] 6
```

```
colnames(cts) %>% length()
```

```
## [1] 6
```

```
all(rownames(coldata) == colnames(cts))
```

```
## [1] TRUE
```

# Create model with DESeq

```
dds <- DESeqDataSetFromMatrix(countData = cts, colData = coldata, design = ~ treatment)

dds <- DESeq(dds)
```

# Transform data (for visualization purposes)

```
myRld <- DESeq2::rlog(dds, blind=FALSE)

myMat <- SummarizedExperiment::assay(myRld)
```

# PCA Function

```
doPCA <- function(pcaData, var_color, var_shape){
    
    percentVar <- round(100 * attr(pcaData, "percentVar"))
    
    g<- ggplot(pcaData, aes_string("PC1", "PC2", color=var_color, shape=var_shape)) +
      geom_point(size=2) +
      xlab(paste0("PC1: ",percentVar[1],"% variance")) +
      ylab(paste0("PC2: ",percentVar[2],"% variance")) + 
      geom_text_repel(data=pcaData,aes(PC1,PC2,label=group), direction="both", nudge_y=0.1, point.padding = 0.6, box.padding=0.25,min.segment.length = unit(0.2, 'lines'),size=2.5) +
      coord_fixed()
    
    return (g)
}
```

# Get PCA data

```
# get PCA data

pcaData <- plotPCA(myRld, intgroup=c("treatment"), returnData=TRUE)
```

# Plot PCA for everything

```
g<- doPCA(pcaData, var_color = "treatment", var_shape = "treatment")

show(g  + scale_color_manual(values=c("blue", "red")) )
```

*Based on this, we can see that mock and cov are quite different*

# DESeq2 degs

```
res = results(dds, contrast= c("treatment", my_exp, my_control) , test="Wald")

summary(res)
```

```
## 
## out of 16818 with nonzero total read count
## adjusted p-value < 0.1
## LFC > 0 (up)       : 3717, 22%
## LFC < 0 (down)     : 3576, 21%
## outliers [1]       : 0, 0%
## low counts [2]     : 2247, 13%
## (mean count < 1)
## [1] see 'cooksCutoff' argument of ?results
## [2] see 'independentFiltering' argument of ?results
```

# Plot top gene

```
geneplot <- function (my_gene, dds, title){
    
    data<- plotCounts(dds, gene=my_gene,intgroup=c("sample", "treatment"), returnData=TRUE)
    
    ggplot(data, aes(x=treatment, y=count, color=treatment, fill=treatment)) +
    scale_y_log10() + 
    geom_dotplot(binaxis='y', stackdir='center') +ggtitle(title) + facet_wrap(~ treatment) + ylab("Normalized Counts\n") +
    theme(plot.title = element_text(hjust = 0.5), 
      axis.title.x=element_blank(),
      axis.title.y=element_text(size=rel(1.5)),
      axis.text.x = element_blank(),
      axis.text.y = element_text(size=rel(1.5)),
      legend.text = element_text(size=rel(1.2)),
      strip.text.x = element_text(size=rel(1.5)),
      axis.ticks.x = element_blank()
      )
}

##

top_genes <- res %>% as.data.frame() %>% tibble::rownames_to_column(var = "gene")%>% arrange(padj) %>% head(n=5) %>% pull(gene)

# Plot for top gene
geneplot(my_gene=top_genes[1], dds=dds, title=top_genes[1])
```

```
## `stat_bindot()` using `bins = 30`. Pick better value with `binwidth`.
```

```
res[top_genes[1],] %>%  kable() %>% kable_styling()
```

|  | baseMean | log2FoldChange | lfcSE | stat | pvalue | padj |
| --- | --- | --- | --- | --- | --- | --- |
| IFIT2 | 12201.58 | 5.29136 | 0.1303285 | 40.60017 | 0 | 0 |

# Volcano Plot

```
doVPlot <- function(results, name){
  
  df <- as.data.frame(results)
  
  df_sig<-subset(df, padj < 0.1)
  
  p <- ggplot(df, aes(log2FoldChange, -log10(pvalue)))  +
    geom_point(size=0.4, color="black", alpha=.8) + 
    geom_point(size=0.4, data=df_sig, aes(log2FoldChange, -log10(pvalue)), colour="red") +
    #xlim(-30,30) +
    #ylim(0, 45) +
    ggtitle(name) +
    theme(
      axis.text.x = element_text(size=12),
      axis.text.y = element_text(size=12),
      axis.title.x = element_text(size=14, margin = margin(t = 10, r = 0, b = 10, l = 0)),
      axis.title.y = element_text(size=14, margin = margin(t = 0, r = 10, b = 0, l = 10)),
      plot.margin =unit(c(.5,.5,.5,.5),"cm"),
      plot.title = element_text(size = 11)
    )
  
  return (p)
}

##

layout <- rbind(c(1,2),c(3,4))


p<- doVPlot(res, paste0(my_exp, " vs. ", my_control))

show(p)
```

```
## Warning: Removed 4979 rows containing missing values (geom_point).
```

# Filter for NAD genes

```
nad <- read.table("nad.genes.txt", sep='\t', header=TRUE)


# check which genes in results


nad_in_results <- res %>% as.data.frame() %>% tibble::rownames_to_column(var = "gene")  %>% pull(gene)

nad_not_in_results <- setdiff(nad$gene, nad_in_results)

nad_not_in_results
```

```
## [1] "NAPRT" "NADK2" "NOCT"
```

```
##
```

*Three NAD genes not present in the download dataset*

# Excel spreadsheets of NAD genes

```
library(writexl)

# excel files based on nad filter

res %>% as.data.frame() %>% rownames_to_column( var = "gene") %>% filter(gene %in% nad$gene)  %>% dplyr::select(-lfcSE,-stat) %>% arrange(padj) %>% write_xlsx(path =paste0(my_series, ".", my_exp, ".vs.", my_control, ".xlsx"))

res %>% as.data.frame() %>% rownames_to_column( var = "gene") %>% filter(gene %in% nad$gene)  %>% dplyr::select(-lfcSE,-stat) %>% arrange(padj) %>% filter(padj < 0.1, abs(log2FoldChange) > 1)  %>%  kable() %>% kable_styling()
```

| gene | baseMean | log2FoldChange | pvalue | padj |
| --- | --- | --- | --- | --- |
| TIPARP | 5401.54594 | 3.093378 | 0.0000000 | 0.0000000 |
| PARP14 | 2649.86312 | 2.976507 | 0.0000000 | 0.0000000 |
| IDO1 | 394.19483 | 4.166600 | 0.0000000 | 0.0000000 |
| ZC3HAV1 | 2512.76760 | 2.427278 | 0.0000000 | 0.0000000 |
| PARP12 | 1665.77468 | 2.297453 | 0.0000000 | 0.0000000 |
| PARP9 | 2354.49173 | 2.449359 | 0.0000000 | 0.0000000 |
| PARP10 | 2376.15625 | 2.092441 | 0.0000000 | 0.0000000 |
| KYNU | 1444.65080 | 1.475528 | 0.0000000 | 0.0000000 |
| NAMPT | 7425.17770 | 1.544902 | 0.0000000 | 0.0000000 |
| PARP8 | 554.25794 | 1.398082 | 0.0000000 | 0.0000000 |
| KMO | 19.54960 | 5.163842 | 0.0000000 | 0.0000000 |
| TNKS2 | 545.87575 | 1.123307 | 0.0000000 | 0.0000001 |
| SLC28A3 | 159.92467 | 1.118403 | 0.0000001 | 0.0000008 |
| SLC25A51 | 45.43648 | 1.180082 | 0.0003904 | 0.0013999 |

*These are the NAD genes with p-adjusted < 0.1 and absolute log 2 fold change > 1.
Others in spreadsheet*

# Session Info

```
sessionInfo()
```

```
## R version 3.5.2 (2018-12-20)
## Platform: x86_64-apple-darwin15.6.0 (64-bit)
## Running under: macOS Mojave 10.14.6
## 
## Matrix products: default
## BLAS: /Library/Frameworks/R.framework/Versions/3.5/Resources/lib/libRblas.0.dylib
## LAPACK: /Library/Frameworks/R.framework/Versions/3.5/Resources/lib/libRlapack.dylib
## 
## locale:
## [1] en_US.UTF-8/en_US.UTF-8/en_US.UTF-8/C/en_US.UTF-8/en_US.UTF-8
## 
## attached base packages:
##  [1] grid      parallel  stats4    stats     graphics  grDevices utils    
##  [8] datasets  methods   base     
## 
## other attached packages:
##  [1] writexl_1.1                 gridExtra_2.3              
##  [3] kableExtra_1.1.0            knitr_1.26                 
##  [5] tibble_2.1.3                ggrepel_0.8.1              
##  [7] ggplot2_3.2.1               DESeq2_1.22.2              
##  [9] SummarizedExperiment_1.12.0 DelayedArray_0.8.0         
## [11] BiocParallel_1.16.6         matrixStats_0.55.0         
## [13] Biobase_2.42.0              GenomicRanges_1.34.0       
## [15] GenomeInfoDb_1.18.2         IRanges_2.16.0             
## [17] S4Vectors_0.20.1            BiocGenerics_0.28.0        
## [19] stringr_1.4.0               dplyr_0.8.3                
## 
## loaded via a namespace (and not attached):
##  [1] bitops_1.0-6           bit64_0.9-7            webshot_0.5.2         
##  [4] RColorBrewer_1.1-2     httr_1.4.1             tools_3.5.2           
##  [7] backports_1.1.5        R6_2.4.1               rpart_4.1-15          
## [10] Hmisc_4.3-0            DBI_1.0.0              lazyeval_0.2.2        
## [13] colorspace_1.4-1       nnet_7.3-12            withr_2.1.2           
## [16] tidyselect_0.2.5       bit_1.1-14             compiler_3.5.2        
## [19] rvest_0.3.4            htmlTable_1.13.3       xml2_1.2.2            
## [22] labeling_0.3           scales_1.1.0           checkmate_1.9.4       
## [25] readr_1.3.1            genefilter_1.64.0      digest_0.6.23         
## [28] foreign_0.8-72         rmarkdown_1.16         XVector_0.22.0        
## [31] base64enc_0.1-3        pkgconfig_2.0.3        htmltools_0.4.0       
## [34] highr_0.8              htmlwidgets_1.5.1      rlang_0.4.2           
## [37] rstudioapi_0.10        RSQLite_2.1.2          farver_2.0.2          
## [40] jsonlite_1.6           acepack_1.4.1          RCurl_1.95-4.12       
## [43] magrittr_1.5           GenomeInfoDbData_1.2.0 Formula_1.2-3         
## [46] Matrix_1.2-17          Rcpp_1.0.3             munsell_0.5.0         
## [49] lifecycle_0.1.0        stringi_1.4.4          yaml_2.2.0            
## [52] zlibbioc_1.28.0        blob_1.2.0             crayon_1.3.4          
## [55] lattice_0.20-38        splines_3.5.2          annotate_1.60.1       
## [58] hms_0.5.3              locfit_1.5-9.1         zeallot_0.1.0         
## [61] pillar_1.4.3           geneplotter_1.60.0     XML_3.98-1.20         
## [64] glue_1.3.1             evaluate_0.14          latticeExtra_0.6-28   
## [67] data.table_1.12.8      vctrs_0.2.1            gtable_0.3.0          
## [70] purrr_0.3.3            assertthat_0.2.1       xfun_0.11             
## [73] xtable_1.8-4           survival_2.44-1.1      viridisLite_0.3.0     
## [76] AnnotationDbi_1.44.0   memoise_1.1.0          cluster_2.1.0
```
