# Supplementary material for: Coronavirus infection and PARP expression dysregulate the NAD metabolome: An actionable component of innate immunity
Source: J Biol Chem. 2021 Jan 13;295(52):17986–96. doi: 10.1074/jbc.RA120.015138 (PMC7834058; doi:10.1074/jbc.RA120.015138)
Supplement: Supplementary file 1 [file mmc1.zip › 161984_1_supp_605519_qh354s.html]

# Brenner RNA-seq Covid 19 Series 16 analysis

#### Henry Keen

#### 04.20.2020

# libraries

```
library(dplyr)
library(stringr)
library(DESeq2)
library(ggplot2)
library(ggrepel)
library(tibble)
library(knitr)
library(kableExtra)
library(grid)
library(gridExtra)
library(writexl)
```

## input parameters

```
my_series  <- "Series16"

my_control <- "Mock"
my_exp   <- "SARS.CoV.2"
my_exp_2 <- "SARS.CoV.2.Rux"
```

# Import data

```
## get counts data

file <- "./04_rawcounts_2020_04_20_modified/GSE147507_RawReadCounts_Human.tsv"

all(file.exists(file))
```

```
## [1] TRUE
```

```
cts <- as.matrix(read.csv(file,sep="\t",row.names="Gene"))


## get metadata

coldata_file <- "./05_coldata_2020_04_20/GSE147507.human.coldata.txt"

coldata <- read.table(coldata_file, sep='\t', header=TRUE)

rownames(coldata) <- coldata$sample


## check
rownames(coldata) %>% length()
```

```
## [1] 78
```

```
colnames(cts) %>% length()
```

```
## [1] 78
```

```
all(rownames(coldata) == colnames(cts))
```

```
## [1] TRUE
```

```
## filter metadata to human data

coldata_original <- coldata

coldata <- coldata %>% filter(series ==  my_series) 

rownames(coldata) <-coldata$sample

## filter counts
cts <- cts[, as.vector(coldata$sample)]

## recheck
cts %>% head()
```

```
##           Series16_A549.ACE2_Mock_1 Series16_A549.ACE2_Mock_2
## DDX11L1                           0                         0
## WASH7P                            0                        11
## FAM138A                           0                         0
## FAM138F                           0                         0
## OR4F5                             0                         0
## LOC729737                         1                         2
##           Series16_A549.ACE2_Mock_3 Series16_A549.ACE2_SARS.CoV.2_1
## DDX11L1                           0                               0
## WASH7P                            7                               2
## FAM138A                           0                               0
## FAM138F                           0                               0
## OR4F5                             0                               0
## LOC729737                         2                               0
##           Series16_A549.ACE2_SARS.CoV.2_2 Series16_A549.ACE2_SARS.CoV.2_3
## DDX11L1                                 0                               0
## WASH7P                                  6                               5
## FAM138A                                 0                               0
## FAM138F                                 0                               0
## OR4F5                                   0                               0
## LOC729737                               0                               1
##           Series16_A549.ACE2_SARS.CoV.2_Rux_1
## DDX11L1                                     0
## WASH7P                                     12
## FAM138A                                     0
## FAM138F                                     0
## OR4F5                                       0
## LOC729737                                   4
##           Series16_A549.ACE2_SARS.CoV.2_Rux_2
## DDX11L1                                     0
## WASH7P                                      6
## FAM138A                                     0
## FAM138F                                     0
## OR4F5                                       0
## LOC729737                                   0
##           Series16_A549.ACE2_SARS.CoV.2_Rux_3
## DDX11L1                                     0
## WASH7P                                      8
## FAM138A                                     0
## FAM138F                                     0
## OR4F5                                       0
## LOC729737                                   2
```

```
coldata %>% head()
```

```
rownames(coldata) %>% length()
```

```
## [1] 9
```

```
colnames(cts) %>% length()
```

```
## [1] 9
```

```
all(rownames(coldata) == colnames(cts))
```

```
## [1] TRUE
```

# Create model with DESeq

```
dds <- DESeqDataSetFromMatrix(countData = cts, colData = coldata, design = ~ treatment)

dds <- DESeq(dds)
```

# Transform data (for visualization purposes)

```
myRld <- DESeq2::rlog(dds, blind=FALSE)

myMat <- SummarizedExperiment::assay(myRld)
```

# PCA Function

```
doPCA <- function(pcaData, var_color, var_shape){
    
    percentVar <- round(100 * attr(pcaData, "percentVar"))
    
    g<- ggplot(pcaData, aes_string("PC1", "PC2", color=var_color, shape=var_shape)) +
      geom_point(size=2) +
      xlab(paste0("PC1: ",percentVar[1],"% variance")) +
      ylab(paste0("PC2: ",percentVar[2],"% variance")) + 
      geom_text_repel(data=pcaData,aes(PC1,PC2,label=group), direction="both", nudge_y=0.1, point.padding = 0.6, box.padding=0.25,min.segment.length = unit(0.2, 'lines'),size=2.5) +
      coord_fixed()
    
    return (g)
}
```

# Get PCA data

```
# get PCA data

pcaData <- plotPCA(myRld, intgroup=c("treatment"), returnData=TRUE)
```

# Plot PCA for everything

```
g<- doPCA(pcaData, var_color = "treatment", var_shape = "treatment")

show(g  + scale_color_manual(values=c("blue", "red", "green")) )
```

*Based on this, we can see that mock is quite different than the other groups*

# DESeq2 degs

```
get_result <- function(my_exp){

  res <- results(dds, contrast= c("treatment", my_exp, my_control) , test="Wald")

  return(res)  
}

##

get_summary<- function(x){
  
  show(x@elementMetadata[2,])
  show(summary(x))
}

##

res <- list()

res <- lapply(c(my_exp,my_exp_2), get_result)

names(res) <- c(my_exp, my_exp_2)

##

lapply(res, get_summary)
```

```
## DataFrame with 1 row and 2 columns
##          type                                          description
##   <character>                                          <character>
## 1     results log2 fold change (MLE): treatment SARS.CoV.2 vs Mock
## 
## out of 17702 with nonzero total read count
## adjusted p-value < 0.1
## LFC > 0 (up)       : 4517, 26%
## LFC < 0 (down)     : 4102, 23%
## outliers [1]       : 1, 0.0056%
## low counts [2]     : 3051, 17%
## (mean count < 1)
## [1] see 'cooksCutoff' argument of ?results
## [2] see 'independentFiltering' argument of ?results
## 
## NULL
## DataFrame with 1 row and 2 columns
##          type                                              description
##   <character>                                              <character>
## 1     results log2 fold change (MLE): treatment SARS.CoV.2.Rux vs Mock
## 
## out of 17702 with nonzero total read count
## adjusted p-value < 0.1
## LFC > 0 (up)       : 4360, 25%
## LFC < 0 (down)     : 4118, 23%
## outliers [1]       : 1, 0.0056%
## low counts [2]     : 3051, 17%
## (mean count < 1)
## [1] see 'cooksCutoff' argument of ?results
## [2] see 'independentFiltering' argument of ?results
## 
## NULL
```

```
## $SARS.CoV.2
## NULL
## 
## $SARS.CoV.2.Rux
## NULL
```

## Plot top gene

```
geneplot <- function (my_gene, dds, title){
    
    data<- plotCounts(dds, gene=my_gene,intgroup=c("sample", "treatment"), returnData=TRUE)
    
    ggplot(data, aes(x=treatment, y=count, color=treatment, fill=treatment)) +
    scale_y_log10() + 
    geom_dotplot(binaxis='y', stackdir='center') +ggtitle(title) + facet_wrap(~ treatment) + ylab("Normalized Counts\n") +
    theme(plot.title = element_text(hjust = 0.5), 
      axis.title.x=element_blank(),
      axis.title.y=element_text(size=rel(1.5)),
      axis.text.x = element_blank(),
      axis.text.y = element_text(size=rel(1.5)),
      legend.text = element_text(size=rel(1.2)),
      strip.text.x = element_text(size=rel(1.5)),
      axis.ticks.x = element_blank()
      )
}

##

plot_top_gene <- function(exp_name,results, my_control){
  
    top_genes <- results[[exp_name]] %>% as.data.frame() %>% tibble::rownames_to_column(var = "gene")%>% arrange(padj) %>% head(n=5) %>% pull(gene)
    
    results[[exp_name]] [top_genes[1],] %>% show
   
    title_name <- paste0(exp_name, " vs. ", my_control, " gene ", top_genes[1])
    
    g<- geneplot(my_gene=top_genes[1], dds=dds, title=title_name)
    
    return(g)
}

##

lapply(names(res), plot_top_gene, results=res, my_control=my_control) %>% show
```

```
## log2 fold change (MLE): treatment SARS.CoV.2 vs Mock 
## Wald test p-value: treatment SARS.CoV.2 vs Mock 
## DataFrame with 1 row and 6 columns
##               baseMean   log2FoldChange             lfcSE             stat
##              <numeric>        <numeric>         <numeric>        <numeric>
## EFNA1 3098.60954179541 4.69399875330681 0.100276557651299 46.8105294323092
##          pvalue      padj
##       <numeric> <numeric>
## EFNA1         0         0
## log2 fold change (MLE): treatment SARS.CoV.2.Rux vs Mock 
## Wald test p-value: treatment SARS.CoV.2.Rux vs Mock 
## DataFrame with 1 row and 6 columns
##               baseMean   log2FoldChange             lfcSE             stat
##              <numeric>        <numeric>         <numeric>        <numeric>
## EFNA1 3098.60954179541 4.60260393017937 0.100099894258675 45.9801078139548
##          pvalue      padj
##       <numeric> <numeric>
## EFNA1         0         0
## [[1]]
```

```
## `stat_bindot()` using `bins = 30`. Pick better value with `binwidth`.
```

```
## 
## [[2]]
```

```
## `stat_bindot()` using `bins = 30`. Pick better value with `binwidth`.
```

# Volcano Plot

```
doVPlot <- function(exp_name, results, my_control){
  
  df <- as.data.frame(results[[exp_name]])
  
  df_sig<-subset(df, padj < 0.1)
  
  title_name <- paste0(exp_name, " vs. ", my_control)
  
  p <- ggplot(df, aes(log2FoldChange, -log10(pvalue)))  +
    geom_point(size=0.4, color="black", alpha=.8) + 
    geom_point(size=0.4, data=df_sig, aes(log2FoldChange, -log10(pvalue)), colour="red") +
    #xlim(-30,30) +
    #ylim(0, 45) +
    ggtitle(title_name) +
    theme(
      axis.text.x = element_text(size=12),
      axis.text.y = element_text(size=12),
      axis.title.x = element_text(size=14, margin = margin(t = 10, r = 0, b = 10, l = 0)),
      axis.title.y = element_text(size=14, margin = margin(t = 0, r = 10, b = 0, l = 10)),
      plot.margin =unit(c(.5,.5,.5,.5),"cm"),
      plot.title = element_text(size = 11)
    )
  
  return (p)
}

##

lapply(names(res), doVPlot, results=res, my_control=my_control) %>% show
```

```
## [[1]]
```

```
## Warning: Removed 4096 rows containing missing values (geom_point).
```

```
## 
## [[2]]
```

```
## Warning: Removed 4096 rows containing missing values (geom_point).
```

# Filter for NAD genes

```
nad <- read.table("nad.genes.txt", sep='\t', header=TRUE)

# check which genes in results

check_nad <- function(res){
  
  nad_in_results <- res %>% as.data.frame() %>% tibble::rownames_to_column(var = "gene")  %>% pull(gene)

  nad_not_in_results <- setdiff(nad$gene, nad_in_results)

  nad_not_in_results 
}

##

lapply(res, check_nad)
```

```
## $SARS.CoV.2
## [1] "NAPRT" "NADK2" "NOCT" 
## 
## $SARS.CoV.2.Rux
## [1] "NAPRT" "NADK2" "NOCT"
```

*Three NAD genes not present in the download dataset*

# Excel spreadsheets of NAD genes

```
# excel files based on nad filter

do_excel <- function(exp_name, results, my_control,nad){
  
  results[[exp_name]] %>% as.data.frame() %>% rownames_to_column( var = "gene") %>% filter(gene %in% nad$gene)  %>% dplyr::select(-lfcSE,-stat) %>% arrange(padj) %>% write_xlsx(path =paste0(my_series, ".", exp_name, ".vs.", my_control, ".xlsx"))
}

lapply(names(res), do_excel, results=res, my_control=my_control,nad=nad)
```

```
## [[1]]
## [1] "/Users/keenhl/Desktop/covid/Series16.SARS.CoV.2.vs.Mock.xlsx"
## 
## [[2]]
## [1] "/Users/keenhl/Desktop/covid/Series16.SARS.CoV.2.Rux.vs.Mock.xlsx"
```

```
# print top results

do_print_top <- function(exp_name, results, my_control,nad){
table <- results[[exp_name]] %>% as.data.frame() %>% rownames_to_column( var = "gene") %>% filter(gene %in% nad$gene)  %>% dplyr::select(-lfcSE,-stat) %>% arrange(padj) %>% filter(padj < 0.1, abs(log2FoldChange) > 1)  

return(table)

}

tables<- lapply(names(res), do_print_top, results=res, my_control=my_control,nad=nad) 

names(tables) <- names(res)
show(tables)
```

```
## $SARS.CoV.2
##        gene    baseMean log2FoldChange       pvalue         padj
## 1    TIPARP 4443.460288       3.747735 0.000000e+00 0.000000e+00
## 2      NQO1 4423.828660      -1.775736 6.809120e-83 5.141939e-81
## 3    PARP14  831.406979       2.468025 1.577416e-79 1.069868e-77
## 4    PARP12  267.092406       2.858475 1.462242e-78 9.737200e-77
## 5     PARP9  353.231503       2.756374 4.410997e-78 2.897807e-76
## 6    PARP10   65.272262       4.425309 2.710007e-47 7.784629e-46
## 7     NAMPT 1967.471171       1.111066 9.984172e-30 1.471293e-28
## 8   ZC3HAV1  835.625386       1.119473 6.160019e-27 7.951037e-26
## 9     PARP1  380.182861      -1.245505 1.738202e-25 2.099313e-24
## 10    PARP6  470.285872       1.119755 7.248020e-22 7.223367e-21
## 11  ADPRHL2  257.173536       1.199970 1.063935e-20 1.004295e-19
## 12    PARP2  125.919175       1.399119 1.141493e-14 7.556655e-14
## 13  SLC29A4   76.551387      -1.857215 5.086297e-14 3.246808e-13
## 14      NNT  102.523369      -1.427900 8.612866e-14 5.401476e-13
## 15     NQO2   97.558249      -1.479771 3.030236e-13 1.820876e-12
## 16  SLC29A1  149.814714      -1.154490 9.996331e-12 5.421927e-11
## 17 SLC25A51  118.303481       1.015152 1.044857e-07 4.069968e-07
## 18  SLC29A3   61.439021      -1.298949 9.845552e-07 3.483152e-06
## 19     BST1   14.134130      -2.274124 1.879773e-05 5.795176e-05
## 20  SLC28A1    5.484184       2.895050 1.873535e-03 4.359803e-03
## 21      KMO   11.862483       1.566872 2.995201e-03 6.723119e-03
## 22     IDO1   13.801731       3.734039 4.314451e-03 9.405759e-03
## 23    NMRK2    1.886247       4.644639 6.042920e-03 1.283026e-02
## 24     QPRT    3.861145      -2.846730 9.391277e-03 1.918729e-02
## 25    PARP8   13.156891       1.414471 1.063019e-02 2.152484e-02
## 
## $SARS.CoV.2.Rux
##       gene    baseMean log2FoldChange       pvalue         padj
## 1   TIPARP 4443.460288       4.098193 0.000000e+00 0.000000e+00
## 2     NQO1 4423.828660      -1.692720 2.079765e-76 1.423764e-74
## 3    PARP6  470.285872       1.135489 4.741035e-23 5.080919e-22
## 4    PARP1  380.182861      -1.046184 5.153269e-20 4.793358e-19
## 5     NQO2   97.558249      -1.528439 4.432769e-15 3.070453e-14
## 6     NNMT  168.173925      -1.605774 6.723297e-15 4.589762e-14
## 7     CD38   56.495892      -2.214410 2.743425e-11 1.452518e-10
## 8    PARP2  125.919175       1.155202 1.296906e-10 6.520136e-10
## 9  SLC29A4   76.551387      -1.415243 3.026361e-10 1.474433e-09
## 10     NNT  102.523369      -1.083136 6.450219e-10 3.058113e-09
## 11    BST1   14.134130      -2.261019 4.964399e-06 1.659709e-05
## 12 SLC29A3   61.439021      -1.112547 9.069133e-06 2.936843e-05
## 13    AOX1   42.377012      -1.257675 1.954117e-05 6.101409e-05
## 14 SLC28A1    5.484184       3.069260 7.374546e-04 1.869801e-03
## 15    QPRT    3.861145      -3.881866 1.988929e-03 4.688305e-03
## 16   NMRK2    1.886247       4.020076 1.839209e-02 3.616215e-02
```

*These are the NAD genes with p-adjusted < 0.1 and absolute log 2 fold change > 1.
Others in spreadsheet*

# Session Info

```
sessionInfo()
```

```
## R version 3.5.2 (2018-12-20)
## Platform: x86_64-apple-darwin15.6.0 (64-bit)
## Running under: macOS Mojave 10.14.6
## 
## Matrix products: default
## BLAS: /Library/Frameworks/R.framework/Versions/3.5/Resources/lib/libRblas.0.dylib
## LAPACK: /Library/Frameworks/R.framework/Versions/3.5/Resources/lib/libRlapack.dylib
## 
## locale:
## [1] en_US.UTF-8/en_US.UTF-8/en_US.UTF-8/C/en_US.UTF-8/en_US.UTF-8
## 
## attached base packages:
##  [1] grid      parallel  stats4    stats     graphics  grDevices utils    
##  [8] datasets  methods   base     
## 
## other attached packages:
##  [1] writexl_1.1                 gridExtra_2.3              
##  [3] kableExtra_1.1.0            knitr_1.26                 
##  [5] tibble_2.1.3                ggrepel_0.8.1              
##  [7] ggplot2_3.2.1               DESeq2_1.22.2              
##  [9] SummarizedExperiment_1.12.0 DelayedArray_0.8.0         
## [11] BiocParallel_1.16.6         matrixStats_0.55.0         
## [13] Biobase_2.42.0              GenomicRanges_1.34.0       
## [15] GenomeInfoDb_1.18.2         IRanges_2.16.0             
## [17] S4Vectors_0.20.1            BiocGenerics_0.28.0        
## [19] stringr_1.4.0               dplyr_0.8.3                
## 
## loaded via a namespace (and not attached):
##  [1] bitops_1.0-6           bit64_0.9-7            webshot_0.5.2         
##  [4] RColorBrewer_1.1-2     httr_1.4.1             tools_3.5.2           
##  [7] backports_1.1.5        R6_2.4.1               rpart_4.1-15          
## [10] Hmisc_4.3-0            DBI_1.0.0              lazyeval_0.2.2        
## [13] colorspace_1.4-1       nnet_7.3-12            withr_2.1.2           
## [16] tidyselect_0.2.5       bit_1.1-14             compiler_3.5.2        
## [19] rvest_0.3.4            htmlTable_1.13.3       xml2_1.2.2            
## [22] labeling_0.3           scales_1.1.0           checkmate_1.9.4       
## [25] readr_1.3.1            genefilter_1.64.0      digest_0.6.23         
## [28] foreign_0.8-72         rmarkdown_1.16         XVector_0.22.0        
## [31] base64enc_0.1-3        pkgconfig_2.0.3        htmltools_0.4.0       
## [34] htmlwidgets_1.5.1      rlang_0.4.2            rstudioapi_0.10       
## [37] RSQLite_2.1.2          farver_2.0.2           jsonlite_1.6          
## [40] acepack_1.4.1          RCurl_1.95-4.12        magrittr_1.5          
## [43] GenomeInfoDbData_1.2.0 Formula_1.2-3          Matrix_1.2-17         
## [46] Rcpp_1.0.3             munsell_0.5.0          lifecycle_0.1.0       
## [49] stringi_1.4.4          yaml_2.2.0             zlibbioc_1.28.0       
## [52] blob_1.2.0             crayon_1.3.4           lattice_0.20-38       
## [55] splines_3.5.2          annotate_1.60.1        hms_0.5.3             
## [58] locfit_1.5-9.1         zeallot_0.1.0          pillar_1.4.3          
## [61] geneplotter_1.60.0     XML_3.98-1.20          glue_1.3.1            
## [64] evaluate_0.14          latticeExtra_0.6-28    data.table_1.12.8     
## [67] vctrs_0.2.1            gtable_0.3.0           purrr_0.3.3           
## [70] assertthat_0.2.1       xfun_0.11              xtable_1.8-4          
## [73] survival_2.44-1.1      viridisLite_0.3.0      AnnotationDbi_1.44.0  
## [76] memoise_1.1.0          cluster_2.1.0
```
