# Supplementary material for: Coronavirus infection and PARP expression dysregulate the NAD metabolome: An actionable component of innate immunity
Source: J Biol Chem. 2021 Jan 13;295(52):17986–96. doi: 10.1074/jbc.RA120.015138 (PMC7834058; doi:10.1074/jbc.RA120.015138)
Supplement: Supplementary file 1 [file mmc1.zip › 161984_1_supp_605520_qh254s.html]

# Brenner RNA-seq Covid 19

#### Henry Keen

#### 04.15.2020

# libraries

```
library(dplyr)
library(stringr)
library(DESeq2)
library(ggplot2)
library(ggrepel)
library(tibble)
library(knitr)
library(kableExtra)
library(grid)
library(gridExtra)
library(writexl)
```

# Import data

```
## get counts data

file <- "./01_rawcounts_2020_04_10_ modified/GSE147507_RawReadCounts_Ferret.tsv"

all(file.exists(file))
```

```
## [1] TRUE
```

```
cts <- as.matrix(read.csv(file,sep="\t",row.names="Gene"))


## get metadata

coldata_file <- "./02_coldata_2020_04_10/GSE147507.ferret.coldata.txt"

coldata <- read.table(coldata_file, sep='\t', header=TRUE)

rownames(coldata) <- coldata$sample


## check

all(rownames(coldata) == colnames(cts))
```

```
## [1] TRUE
```

# Create model with DESeq

```
dds <- DESeqDataSetFromMatrix(countData = cts, colData = coldata, design = ~ cell + treatment + time)

dds <- dds[ rowSums(counts(dds)) > 10, ]

dds <- DESeq(dds)
```

# Transform data (for visualization purposes)

```
myRld <- DESeq2::rlog(dds, blind=FALSE)

myMat <- SummarizedExperiment::assay(myRld)
```

# PCA Function

```
doPCA <- function(pcaData, var_color, var_shape){
    
    percentVar <- round(100 * attr(pcaData, "percentVar"))
    
    g<- ggplot(pcaData, aes_string("PC1", "PC2", color=var_color, shape=var_shape)) +
      geom_point(size=2) +
      xlab(paste0("PC1: ",percentVar[1],"% variance")) +
      ylab(paste0("PC2: ",percentVar[2],"% variance")) + 
      geom_text_repel(data=pcaData,aes(PC1,PC2,label=group), direction="both", nudge_y=0.1, point.padding = 0.6, box.padding=0.25,min.segment.length = unit(0.2, 'lines'),size=2.5) +
      coord_fixed()
    
    return (g)
}
```

# Get PCA data

```
# get PCA data

pcaData <- plotPCA(myRld, intgroup=c("cell", "treatment", "time"), returnData=TRUE)
```

# Plot PCA for everything

```
g<- doPCA(pcaData, var_color = "cell", var_shape = "treatment")

show(g  + scale_color_manual(values=c("blue", "red")) )
```

*Based on this, we can see that 1)trachea and NW are clearly different, 2) day 14 in
the NW samples is different compared to other days. At this view, itâ€™s too difficult
to discern all the points on the top.*

# Plot PCA for trachea

```
## subset for trachea only

trachea <- pcaData %>% filter(cell=='FerretTrachea')

g<- doPCA(trachea,var_color = "treatment", var_shape = "time")

show(g)
```

*There does seem to be some difference between Cov relative to other samples in the
trachea*

# Plot PCA for NW

```
## subset for NW only not including day14

NW <- pcaData %>% filter(cell=='FerretNW', time != 'd14')

g<- doPCA(NW,var_color = "treatment", var_shape = "time")

show(g + facet_grid( ~ time    ))
```

*The difference between Cov and other samples seems to increase with time. IAV seems
to have bigger effect than Cov in the day 7 data*

# Annotation for ferret

```
library(biomaRt)

get_annotation <- function(biomart_dataset, idtype){
  if(is.null(biomart_dataset))
    stop("Select a species to generate the corresponding annotation.
         To obtain a list, type mart = useMart('ensembl'), followed by listDatasets(mart).")
  
  mart <- useMart(biomart="ENSEMBL_MART_ENSEMBL",
                  host="www.ensembl.org",
                  dataset=biomart_dataset)
  
  anns <- getBM(attributes = c(idtype, "external_gene_name", "description"),
                filters = idtype,
                values= "",
                mart = mart)
  
  return(anns)
}

## get annotation

anno_file_name <- "ferret.anno.rds"

## check if annotation already downloaded 

if (file.exists(anno_file_name)){
  
  anno <- readRDS(anno_file_name)
} else{

  anno <- get_annotation('mpfuro_gene_ensembl','ensembl_gene_id')
  anno <- na.omit(anno)

  rownames(anno) <- anno$ensembl_gene_id

  saveRDS(anno, anno_file_name)
}

# function to add annotation to results

add_anno <- function(res1, anno){

  res1$gene                     <- rownames(res1)
  res1$ext_gene                 <- anno[row.names(res1), "external_gene_name"]
  res1$ext_gene_description  <- anno[row.names(res1), "description"]
    
  return(res1)  
}
```

## Split up samples â€" Trachea

```
# create list objects: metatdata, counts, deseq object

mcoldata <- list()
mcts <- list()
mdds <- list()
```

# Postive control genes

```
IFNB1 <- "ENSMPUG00000019528"
IL6 <- "ENSMPUG00000013592"
```

# DESeq2 for trachea

```
# short name for list in this chunk

m <- "tr"


# filter metadata to trachea data

mcoldata[[m]] <- coldata %>% filter(cell == "FerretTrachea") 

rownames(mcoldata[[m]]) <- mcoldata[[m]]$sample


# filter counts to trachea samples

mcts[[m]] <- cts[, as.vector(mcoldata[[m]]$sample)]


## check

all(rownames(mcoldata[[m]]) == colnames(mcts[[m]]))
```

```
## [1] TRUE
```

```
##

mdds[[m]] <- DESeqDataSetFromMatrix(countData = mcts[[m]], colData = mcoldata[[m]], design = ~ treatment)

mdds[[m]] <- DESeq(mdds[[m]])
```

```
## estimating size factors
```

```
## estimating dispersions
```

```
## gene-wise dispersion estimates
```

```
## mean-dispersion relationship
```

```
## final dispersion estimates
```

```
## fitting model and testing
```

```
##

res_trachea_cov_ctl = results(mdds[[m]], contrast= c("treatment", "SARS.CoV.2", "Ctl") , test="Wald") %>% add_anno(anno = anno)

summary(res_trachea_cov_ctl)
```

```
## 
## out of 23308 with nonzero total read count
## adjusted p-value < 0.1
## LFC > 0 (up)       : 4172, 18%
## LFC < 0 (down)     : 4223, 18%
## outliers [1]       : 95, 0.41%
## low counts [2]     : 7073, 30%
## (mean count < 1)
## [1] see 'cooksCutoff' argument of ?results
## [2] see 'independentFiltering' argument of ?results
```

```
##

res_trachea_iav_ctl = results(mdds[[m]], contrast= c("treatment", "IAV", "Ctl") , test="Wald") %>% add_anno(anno = anno)

summary(res_trachea_iav_ctl)
```

```
## 
## out of 23308 with nonzero total read count
## adjusted p-value < 0.1
## LFC > 0 (up)       : 3101, 13%
## LFC < 0 (down)     : 3856, 17%
## outliers [1]       : 95, 0.41%
## low counts [2]     : 7511, 32%
## (mean count < 2)
## [1] see 'cooksCutoff' argument of ?results
## [2] see 'independentFiltering' argument of ?results
```

```
# Positive control genes

# Cov vs. control
res_trachea_cov_ctl[IFNB1, ] %>%  kable() %>% kable_styling()
```

|  | baseMean | log2FoldChange | lfcSE | stat | pvalue | padj | gene | ext\_gene | ext\_gene\_description |
| --- | --- | --- | --- | --- | --- | --- | --- | --- | --- |
| ENSMPUG00000019528 | 0.0597451 | -0.4073305 | 4.046693 | -0.1006576 | 0.9198223 | NA | ENSMPUG00000019528 | IFNB1 | Mustela putorius furo interferon beta 1 (IFNB1), mRNA. [Source:RefSeq mRNA;Acc:NM\_001310212] |

```
res_trachea_cov_ctl[IL6, ] %>% kable() %>% kable_styling()
```

|  | baseMean | log2FoldChange | lfcSE | stat | pvalue | padj | gene | ext\_gene | ext\_gene\_description |
| --- | --- | --- | --- | --- | --- | --- | --- | --- | --- |
| ENSMPUG00000013592 | 4.151559 | -0.9886945 | 1.164826 | -0.8487913 | 0.3959974 | 0.5029528 | ENSMPUG00000013592 | IL6 | Interleukin-6 [Source:UniProtKB/Swiss-Prot;Acc:A3FBE9] |

```
# IAV vs. control
res_trachea_iav_ctl[IFNB1, ] %>% kable() %>% kable_styling()
```

|  | baseMean | log2FoldChange | lfcSE | stat | pvalue | padj | gene | ext\_gene | ext\_gene\_description |
| --- | --- | --- | --- | --- | --- | --- | --- | --- | --- |
| ENSMPUG00000019528 | 0.0597451 | 0 | 3.687917 | 0 | 1 | NA | ENSMPUG00000019528 | IFNB1 | Mustela putorius furo interferon beta 1 (IFNB1), mRNA. [Source:RefSeq mRNA;Acc:NM\_001310212] |

```
res_trachea_iav_ctl[IL6, ] %>% kable() %>% kable_styling()
```

|  | baseMean | log2FoldChange | lfcSE | stat | pvalue | padj | gene | ext\_gene | ext\_gene\_description |
| --- | --- | --- | --- | --- | --- | --- | --- | --- | --- |
| ENSMPUG00000013592 | 4.151559 | -2.203708 | 1.173587 | -1.877755 | 0.0604147 | 0.1255303 | ENSMPUG00000013592 | IL6 | Interleukin-6 [Source:UniProtKB/Swiss-Prot;Acc:A3FBE9] |

# DESeq2 for nw

```
# short name for list in this chunk

m <- "nw"


# filter metadata to nw data
# exclude day 1

mcoldata[[m]] <- coldata %>% filter(cell == "FerretNW", time != 'd1')

rownames(mcoldata[[m]]) <- mcoldata[[m]]$sample


# filter counts to nw samples

mcts[[m]] <- cts[, as.vector(mcoldata[[m]]$sample)]


## check

all(rownames(mcoldata[[m]]) == colnames(mcts[[m]]))
```

```
## [1] TRUE
```

```
##

mdds[[m]] <- DESeqDataSetFromMatrix(countData = mcts[[m]], colData = mcoldata[[m]], design = ~ treatment + time)
```

```
## factor levels were dropped which had no samples
```

```
mdds[[m]] <- DESeq(mdds[[m]])
```

```
## estimating size factors
```

```
## estimating dispersions
```

```
## gene-wise dispersion estimates
```

```
## mean-dispersion relationship
```

```
## final dispersion estimates
```

```
## fitting model and testing
```

```
##

res_nw_cov_ctl <- results(mdds[[m]], contrast= c("treatment", "SARS.CoV.2", "Ctl") , test="Wald") %>% add_anno(anno = anno)

summary(res_nw_cov_ctl)
```

```
## 
## out of 22762 with nonzero total read count
## adjusted p-value < 0.1
## LFC > 0 (up)       : 5, 0.022%
## LFC < 0 (down)     : 14, 0.062%
## outliers [1]       : 0, 0%
## low counts [2]     : 13386, 59%
## (mean count < 25)
## [1] see 'cooksCutoff' argument of ?results
## [2] see 'independentFiltering' argument of ?results
```

```
##

res_nw_iav_ctl <- results(mdds[[m]], contrast= c("treatment", "IAV", "Ctl") , test="Wald") %>% add_anno(anno = anno)

summary(res_nw_iav_ctl)
```

```
## 
## out of 22762 with nonzero total read count
## adjusted p-value < 0.1
## LFC > 0 (up)       : 1894, 8.3%
## LFC < 0 (down)     : 1405, 6.2%
## outliers [1]       : 0, 0%
## low counts [2]     : 9068, 40%
## (mean count < 4)
## [1] see 'cooksCutoff' argument of ?results
## [2] see 'independentFiltering' argument of ?results
```

```
# Postive control genes

# Cov vs. control
res_nw_cov_ctl[IFNB1, ] %>%  kable() %>% kable_styling()
```

|  | baseMean | log2FoldChange | lfcSE | stat | pvalue | padj | gene | ext\_gene | ext\_gene\_description |
| --- | --- | --- | --- | --- | --- | --- | --- | --- | --- |
| ENSMPUG00000019528 | 15.22714 | 1.204664 | 3.235672 | 0.3723072 | 0.7096641 | NA | ENSMPUG00000019528 | IFNB1 | Mustela putorius furo interferon beta 1 (IFNB1), mRNA. [Source:RefSeq mRNA;Acc:NM\_001310212] |

```
res_nw_cov_ctl[IL6, ] %>%  kable() %>% kable_styling()
```

|  | baseMean | log2FoldChange | lfcSE | stat | pvalue | padj | gene | ext\_gene | ext\_gene\_description |
| --- | --- | --- | --- | --- | --- | --- | --- | --- | --- |
| ENSMPUG00000013592 | 36.97506 | -0.6296351 | 0.883653 | -0.7125366 | 0.4761326 | 0.942718 | ENSMPUG00000013592 | IL6 | Interleukin-6 [Source:UniProtKB/Swiss-Prot;Acc:A3FBE9] |

```
# IAV vs. control
res_nw_iav_ctl[IFNB1, ] %>%  kable() %>% kable_styling()
```

|  | baseMean | log2FoldChange | lfcSE | stat | pvalue | padj | gene | ext\_gene | ext\_gene\_description |
| --- | --- | --- | --- | --- | --- | --- | --- | --- | --- |
| ENSMPUG00000019528 | 15.22714 | 3.644955 | 5.245544 | 0.6948669 | 0.4871387 | 0.6978918 | ENSMPUG00000019528 | IFNB1 | Mustela putorius furo interferon beta 1 (IFNB1), mRNA. [Source:RefSeq mRNA;Acc:NM\_001310212] |

```
res_nw_iav_ctl[IL6, ] %>%  kable() %>% kable_styling()
```

|  | baseMean | log2FoldChange | lfcSE | stat | pvalue | padj | gene | ext\_gene | ext\_gene\_description |
| --- | --- | --- | --- | --- | --- | --- | --- | --- | --- |
| ENSMPUG00000013592 | 36.97506 | -1.557721 | 1.423599 | -1.094213 | 0.2738613 | 0.5004346 | ENSMPUG00000013592 | IL6 | Interleukin-6 [Source:UniProtKB/Swiss-Prot;Acc:A3FBE9] |

# Plot positive control genes

```
geneplot <- function (my_gene, dds, title){
    
    data<- plotCounts(dds, gene=my_gene,intgroup=c("sample", "cell", "treatment", "time" ), returnData=TRUE)
    
    data$time <- factor(data$time, levels=c("d1", "d3", "d7", "d14"))
    
    ggplot(data, aes(x=treatment, y=count, color=treatment, fill=treatment)) +
    scale_y_log10() + 
    geom_dotplot(binaxis='y', stackdir='center') +ggtitle(title) + facet_wrap(~ cell + time) + ylab("Normalized Counts\n") +
    theme(plot.title = element_text(hjust = 0.5), 
      axis.title.x=element_blank(),
      axis.title.y=element_text(size=rel(1.5)),
      axis.text.x = element_blank(),
      axis.text.y = element_text(size=rel(1.5)),
      legend.text = element_text(size=rel(1.2)),
      strip.text.x = element_text(size=rel(1.5)),
      axis.ticks.x = element_blank()
      )
}

##

# Plot for IL6
geneplot(my_gene=IL6, dds=dds, title="IL6")
```

```
## `stat_bindot()` using `bins = 30`. Pick better value with `binwidth`.
```

```
# Plot for IFNB1
geneplot(my_gene=IFNB1, dds=dds, title="IFNB1")
```

```
## `stat_bindot()` using `bins = 30`. Pick better value with `binwidth`.
```

```
# Plot for top NAD set degs
geneplot(my_gene="ENSMPUG00000003685", dds=dds, title="KYNU")
```

```
## `stat_bindot()` using `bins = 30`. Pick better value with `binwidth`.
```

*The positive controls are inconsisent but are, in fact, considered to not be very
good positive controls. Just ignore.*

# Volcano Plot

```
doVPlot <- function(name, results){
  
  df <- as.data.frame(results[[name]])
  
  df_sig<-subset(df, padj < 0.1)
  
  p <- ggplot(df, aes(log2FoldChange, -log10(pvalue)))  +
    geom_point(size=0.4, color="black", alpha=.8) + 
    geom_point(size=0.4, data=df_sig, aes(log2FoldChange, -log10(pvalue)), colour="red") +
    #xlim(-30,30) +
    #ylim(0, 45) +
    ggtitle(name) +
    theme(
      axis.text.x = element_text(size=12),
      axis.text.y = element_text(size=12),
      axis.title.x = element_text(size=14, margin = margin(t = 10, r = 0, b = 10, l = 0)),
      axis.title.y = element_text(size=14, margin = margin(t = 0, r = 10, b = 0, l = 10)),
      plot.margin =unit(c(.5,.5,.5,.5),"cm"),
      plot.title = element_text(size = 11)
    )
  
  return (p)
}

##

results <- list()

results[[" Trachea - SARS-CoV-2 vs. control "]] <- res_trachea_cov_ctl 
results[[" Trachea - IAV vs. control "]] <- res_trachea_iav_ctl

results[[" NW - SARS-CoV-2 vs. control "]] <- res_nw_cov_ctl 
results[[" NW - IAV vs. control "]] <- res_nw_iav_ctl 

##

layout <- rbind(c(1,2),c(3,4))

p <- list()

p<- lapply(names(results), doVPlot, results=results)

p[[1]] <- p[[1]] + xlim(-10,10) + ylim(0,40)
p[[2]] <- p[[2]] + xlim(-10,10) + ylim(0,40)

p[[3]] <- p[[3]] + xlim(-30,30) + ylim(0,30)
p[[4]] <- p[[4]] + xlim(-30,30) + ylim(0,30)

##

marrangeGrob(grobs=p, nrow=2, ncol=2, layout_matrix=layout, top = textGrob("\n",gp=gpar(fontsize=16)))
```

```
## Warning: Removed 8845 rows containing missing values (geom_point).
```

```
## Warning: Removed 1 rows containing missing values (geom_point).
```

```
## Warning: Removed 8844 rows containing missing values (geom_point).
```

```
## Warning: Removed 9295 rows containing missing values (geom_point).

## Warning: Removed 9295 rows containing missing values (geom_point).
```

# Filter for NAD genes

```
nad <- read.table("nad.genes.txt", sep='\t', header=TRUE)


# check which genes in annotation

nad_in_anno <- anno %>% filter(external_gene_name %in% nad$gene) %>% pull(external_gene_name)

nad_not_in_anno <- setdiff(nad$gene, nad_in_anno)

nad_not_in_anno
```

```
## [1] "NADSYN1"  "NMRK2"    "NADK"     "PARP10"   "SIRT7"    "CD38"     "SLC28A2" 
## [8] "SLC25A51" "SLC25A52"
```

```
##
```

# Excel spreadsheets of NAD genes

```
# excel files based on nad filter

res_trachea_cov_ctl %>% as.data.frame() %>% filter(ext_gene %in% nad$gene)  %>% dplyr::select(-lfcSE,-stat) %>% arrange(padj) %>% write_xlsx(path ="nad.trachea.cov.vs.ctl.xlsx")
res_trachea_iav_ctl %>% as.data.frame() %>% filter(ext_gene %in% nad$gene)  %>% dplyr::select(-lfcSE,-stat) %>% arrange(padj) %>% write_xlsx(path ="nad.trachea.iav.vs.ctl.xlsx")

res_nw_cov_ctl %>% as.data.frame() %>% filter(ext_gene %in% nad$gene)  %>% dplyr::select(-lfcSE,-stat) %>% arrange(padj) %>% write_xlsx(path ="nad.nw.cov.vs.ctl.xlsx")
res_nw_iav_ctl %>% as.data.frame() %>% filter(ext_gene %in% nad$gene)  %>% dplyr::select(-lfcSE,-stat) %>% arrange(padj) %>% write_xlsx(path ="nad.nw.iav.vs.ctl.xlsx")
```

# Volcano plots based on NAD data

```
doVPlot_2 <- function(name, results, my_filter){
  
  df <- as.data.frame(results[[name]])
  
  df_sig<-subset(df, padj < 0.1)
  
  df_filter<- df %>% filter(ext_gene %in% my_filter$gene, padj <= 0.1) %>% arrange(padj)
  
  df_filter_label<- df %>% filter(ext_gene %in% my_filter$gene, padj <= 0.1, log2FoldChange > 0) %>% arrange(padj) %>% head(n=5)
  
  p <- ggplot(df, aes(log2FoldChange, -log10(pvalue)))  +
    geom_point(size=0.4, color="black", alpha=.6) + 
    geom_point(size=0.4, alpha=0.6, data=df_sig, aes(log2FoldChange, -log10(pvalue)), colour="red") +
    geom_point(size=0.4, data=df_filter, aes(log2FoldChange, -log10(pvalue)), colour="blue") +
    #xlim(-30,30) +
    #ylim(0, 45) +
    ggtitle(name) +
    geom_text_repel(size= 1.2, 
                    colour="black",
                    #xlim=c(4,NA),
                    ylim=c(6,NA),
                    segment.size=0.1, 
                    nudge_x=2, 
                    nudge_y=12, 
                    data=df_filter_label,
                    aes(log2FoldChange, -log10(pvalue), label=ext_gene), 
                    max.iter= 20000, 
                    point.padding = 0.15, 
                    segment.alpha = 1, 
                    box.padding=.15,
                    min.segment.length = unit(0.15, 'lines'),size=2.5) +
   theme(
      axis.text.x = element_text(size=12),
      axis.text.y = element_text(size=12),
      axis.title.x = element_text(size=14, margin = margin(t = 10, r = 0, b = 10, l = 0)),
      axis.title.y = element_text(size=14, margin = margin(t = 0, r = 10, b = 0, l = 10)),
      plot.margin =unit(c(.5,.5,.5,.5),"cm"),
      plot.title = element_text(size = 11)
    )
  
  return (p)
}

##

layout <- rbind(c(1,2),c(3,4))

p <- list()

p<- lapply(names(results), doVPlot_2, results=results, my_filter=nad)
```

```
## Warning: Duplicated aesthetics after name standardisation: size

## Warning: Duplicated aesthetics after name standardisation: size

## Warning: Duplicated aesthetics after name standardisation: size

## Warning: Duplicated aesthetics after name standardisation: size
```

```
p[[1]] <- p[[1]] + xlim(-10,10) + ylim(0,40)
p[[2]] <- p[[2]] + xlim(-10,10) + ylim(0,40)

p[[3]] <- p[[3]] + xlim(-30,30) + ylim(0,30)
p[[4]] <- p[[4]] + xlim(-30,30) + ylim(0,30)

##

marrangeGrob(grobs=p, nrow=2, ncol=2, layout_matrix=layout, top = textGrob("\n",gp=gpar(fontsize=16)))
```

```
## Warning: Removed 8845 rows containing missing values (geom_point).
```

```
## Warning: Removed 1 rows containing missing values (geom_point).
```

```
## Warning: Removed 8844 rows containing missing values (geom_point).
```

```
## Warning: Removed 9295 rows containing missing values (geom_point).

## Warning: Removed 9295 rows containing missing values (geom_point).
```

\* NAD genes with adjusted p-value < 0.1 are highlighted in blue. The labeled genes
are the top 5 upregulated NAD genes, based on adjused p-value (note, in a previous
version of this file the labels highlighted 5 of the differentially expressed NAD
genes but not the top 5, corrected by added arrange and head to the code) \*

# Session Info

```
sessionInfo()
```

```
## R version 3.5.2 (2018-12-20)
## Platform: x86_64-apple-darwin15.6.0 (64-bit)
## Running under: macOS Mojave 10.14.6
## 
## Matrix products: default
## BLAS: /Library/Frameworks/R.framework/Versions/3.5/Resources/lib/libRblas.0.dylib
## LAPACK: /Library/Frameworks/R.framework/Versions/3.5/Resources/lib/libRlapack.dylib
## 
## locale:
## [1] en_US.UTF-8/en_US.UTF-8/en_US.UTF-8/C/en_US.UTF-8/en_US.UTF-8
## 
## attached base packages:
##  [1] grid      parallel  stats4    stats     graphics  grDevices utils    
##  [8] datasets  methods   base     
## 
## other attached packages:
##  [1] biomaRt_2.38.0              writexl_1.1                
##  [3] gridExtra_2.3               kableExtra_1.1.0           
##  [5] knitr_1.26                  tibble_2.1.3               
##  [7] ggrepel_0.8.1               ggplot2_3.2.1              
##  [9] DESeq2_1.22.2               SummarizedExperiment_1.12.0
## [11] DelayedArray_0.8.0          BiocParallel_1.16.6        
## [13] matrixStats_0.55.0          Biobase_2.42.0             
## [15] GenomicRanges_1.34.0        GenomeInfoDb_1.18.2        
## [17] IRanges_2.16.0              S4Vectors_0.20.1           
## [19] BiocGenerics_0.28.0         stringr_1.4.0              
## [21] dplyr_0.8.3                
## 
## loaded via a namespace (and not attached):
##  [1] bitops_1.0-6           bit64_0.9-7            progress_1.2.2        
##  [4] webshot_0.5.2          RColorBrewer_1.1-2     httr_1.4.1            
##  [7] tools_3.5.2            backports_1.1.5        R6_2.4.1              
## [10] rpart_4.1-15           Hmisc_4.3-0            DBI_1.0.0             
## [13] lazyeval_0.2.2         colorspace_1.4-1       nnet_7.3-12           
## [16] withr_2.1.2            prettyunits_1.0.2      tidyselect_0.2.5      
## [19] bit_1.1-14             compiler_3.5.2         rvest_0.3.4           
## [22] htmlTable_1.13.3       xml2_1.2.2             labeling_0.3          
## [25] scales_1.1.0           checkmate_1.9.4        readr_1.3.1           
## [28] genefilter_1.64.0      digest_0.6.23          foreign_0.8-72        
## [31] rmarkdown_1.16         XVector_0.22.0         base64enc_0.1-3       
## [34] pkgconfig_2.0.3        htmltools_0.4.0        highr_0.8             
## [37] htmlwidgets_1.5.1      rlang_0.4.2            rstudioapi_0.10       
## [40] RSQLite_2.1.2          farver_2.0.2           acepack_1.4.1         
## [43] RCurl_1.95-4.12        magrittr_1.5           GenomeInfoDbData_1.2.0
## [46] Formula_1.2-3          Matrix_1.2-17          Rcpp_1.0.3            
## [49] munsell_0.5.0          lifecycle_0.1.0        stringi_1.4.4         
## [52] yaml_2.2.0             zlibbioc_1.28.0        plyr_1.8.5            
## [55] blob_1.2.0             crayon_1.3.4           lattice_0.20-38       
## [58] splines_3.5.2          annotate_1.60.1        hms_0.5.3             
## [61] locfit_1.5-9.1         zeallot_0.1.0          pillar_1.4.3          
## [64] reshape2_1.4.3         geneplotter_1.60.0     XML_3.98-1.20         
## [67] glue_1.3.1             evaluate_0.14          latticeExtra_0.6-28   
## [70] data.table_1.12.8      vctrs_0.2.1            gtable_0.3.0          
## [73] purrr_0.3.3            assertthat_0.2.1       xfun_0.11             
## [76] xtable_1.8-4           survival_2.44-1.1      viridisLite_0.3.0     
## [79] AnnotationDbi_1.44.0   memoise_1.1.0          cluster_2.1.0
```
