# Supplementary material for: Coronavirus infection and PARP expression dysregulate the NAD metabolome: An actionable component of innate immunity
Source: J Biol Chem. 2021 Jan 13;295(52):17986–96. doi: 10.1074/jbc.RA120.015138 (PMC7834058; doi:10.1074/jbc.RA120.015138)
Supplement: Supplementary file 1 [file mmc1.zip › 161984_1_supp_605521_qhj54s.html]

# Brenner RNA-seq Covid 19

#### Henry Keen

#### 04.13.2020

# libraries

```
library(dplyr)
library(stringr)
library(DESeq2)
library(ggplot2)
library(ggrepel)
library(tibble)
library(knitr)
library(kableExtra)
library(grid)
library(gridExtra)
```

# Import data

```
## get counts data

file <- "./01_rawcounts_2020_04_10_ modified/GSE147507_RawReadCounts_Human.tsv"

all(file.exists(file))
```

```
## [1] TRUE
```

```
cts <- as.matrix(read.csv(file,sep="\t",row.names="Gene"))


## get metadata

coldata_file <- "./02_coldata_2020_04_10/GSE147507.human.coldata.txt"

coldata <- read.table(coldata_file, sep='\t', header=TRUE)

rownames(coldata) <- coldata$sample


## check
all(rownames(coldata) == colnames(cts))
```

```
## [1] TRUE
```

```
## filter metadata to human data
coldata <- coldata %>% filter(series == "Series15") 

rownames(coldata) <-coldata$sample

## filter counts
cts <- cts[, as.vector(coldata$sample)]

## check
cts %>% head()
```

```
##           Series15_HealthyLungBiopsy_2 Series15_HealthyLungBiopsy_1
## DDX11L1                              0                            0
## WASH7P                             261                          140
## FAM138A                              0                            0
## FAM138F                              0                            0
## OR4F5                                0                            0
## LOC729737                           15                           70
##           Series15_COVID19Lung_2 Series15_COVID19Lung_1
## DDX11L1                        0                      0
## WASH7P                         0                      0
## FAM138A                        0                      0
## FAM138F                        0                      0
## OR4F5                          0                      0
## LOC729737                     17                      0
```

```
coldata %>% head()
```

```
all(rownames(coldata) == colnames(cts))
```

```
## [1] TRUE
```

# Create model with DESeq

```
dds <- DESeqDataSetFromMatrix(countData = cts, colData = coldata, design = ~ cell)

dds <- DESeq(dds)
```

# Transform data (for visualization purposes)

```
myRld <- DESeq2::rlog(dds, blind=FALSE)

myMat <- SummarizedExperiment::assay(myRld)
```

# PCA Function

```
doPCA <- function(pcaData, var_color, var_shape){
    
    percentVar <- round(100 * attr(pcaData, "percentVar"))
    
    g<- ggplot(pcaData, aes_string("PC1", "PC2", color=var_color, shape=var_shape)) +
      geom_point(size=2) +
      xlab(paste0("PC1: ",percentVar[1],"% variance")) +
      ylab(paste0("PC2: ",percentVar[2],"% variance")) + 
      geom_text_repel(data=pcaData,aes(PC1,PC2,label=group), direction="both", nudge_y=0.1, point.padding = 0.6, box.padding=0.25,min.segment.length = unit(0.2, 'lines'),size=2.5) +
      coord_fixed()
    
    return (g)
}
```

# Get PCA data

```
# get PCA data

pcaData <- plotPCA(myRld, intgroup=c("cell"), returnData=TRUE)
```

# Plot PCA for everything

```
g<- doPCA(pcaData, var_color = "cell", var_shape = "cell")

show(g  + scale_color_manual(values=c("blue", "red")) )
```

*Based on this, we can see that healthy lung and cov lung are quite different*

# Annotation for human

```
library(biomaRt)

get_annotation <- function(biomart_dataset, idtype){
  if(is.null(biomart_dataset))
    stop("Select a species to generate the corresponding annotation.
         To obtain a list, type mart = useMart('ensembl'), followed by listDatasets(mart).")
  
  mart <- useMart(biomart="ENSEMBL_MART_ENSEMBL",
                  host="www.ensembl.org",
                  dataset=biomart_dataset)
  
  anns <- getBM(attributes = c(idtype, "external_gene_name", "description"),
                filters = idtype,
                values= "",
                mart = mart)
  
  return(anns)
}

## get annotation

anno_file_name <- "human.anno.rds"

## check if annotation already downloaded 

if (file.exists(anno_file_name)){
  
  anno <- readRDS(anno_file_name)
} else{

  anno <- get_annotation('hsapiens_gene_ensembl','ensembl_gene_id')
  anno <- na.omit(anno)

  rownames(anno) <- anno$ensembl_gene_id

  saveRDS(anno, anno_file_name)
}
```

# DESeq2 degs

```
res_human = results(dds, contrast= c("cell", "COVID19Lung", "HealthyLungBiopsy") , test="Wald")

summary(res_human)
```

```
## 
## out of 18281 with nonzero total read count
## adjusted p-value < 0.1
## LFC > 0 (up)       : 505, 2.8%
## LFC < 0 (down)     : 891, 4.9%
## outliers [1]       : 0, 0%
## low counts [2]     : 8771, 48%
## (mean count < 15)
## [1] see 'cooksCutoff' argument of ?results
## [2] see 'independentFiltering' argument of ?results
```

# Plot top gene

```
geneplot <- function (my_gene, dds, title){
    
    data<- plotCounts(dds, gene=my_gene,intgroup=c("sample", "cell"), returnData=TRUE)
    
    ggplot(data, aes(x=cell, y=count, color=cell, fill=cell)) +
    scale_y_log10() + 
    geom_dotplot(binaxis='y', stackdir='center') +ggtitle(title) + facet_wrap(~ cell) + ylab("Normalized Counts\n") +
    theme(plot.title = element_text(hjust = 0.5), 
      axis.title.x=element_blank(),
      axis.title.y=element_text(size=rel(1.5)),
      axis.text.x = element_blank(),
      axis.text.y = element_text(size=rel(1.5)),
      legend.text = element_text(size=rel(1.2)),
      strip.text.x = element_text(size=rel(1.5)),
      axis.ticks.x = element_blank()
      )
}

##

top_genes <- res_human %>% as.data.frame() %>% tibble::rownames_to_column(var = "gene")%>% arrange(padj) %>% head(n=5) %>% pull(gene)

# Plot for top gene
geneplot(my_gene=top_genes[1], dds=dds, title=top_genes[1])
```

```
## `stat_bindot()` using `bins = 30`. Pick better value with `binwidth`.
```

```
res_human[top_genes[1],] %>%  kable() %>% kable_styling()
```

|  | baseMean | log2FoldChange | lfcSE | stat | pvalue | padj |
| --- | --- | --- | --- | --- | --- | --- |
| SELL | 10726.93 | 8.792633 | 0.8994143 | 9.775954 | 0 | 0 |

# Volcano Plot

```
doVPlot <- function(results, name){
  
  df <- as.data.frame(results)
  
  df_sig<-subset(df, padj < 0.1)
  
  p <- ggplot(df, aes(log2FoldChange, -log10(pvalue)))  +
    geom_point(size=0.4, color="black", alpha=.8) + 
    geom_point(size=0.4, data=df_sig, aes(log2FoldChange, -log10(pvalue)), colour="red") +
    #xlim(-30,30) +
    #ylim(0, 45) +
    ggtitle(name) +
    theme(
      axis.text.x = element_text(size=12),
      axis.text.y = element_text(size=12),
      axis.title.x = element_text(size=14, margin = margin(t = 10, r = 0, b = 10, l = 0)),
      axis.title.y = element_text(size=14, margin = margin(t = 0, r = 10, b = 0, l = 10)),
      plot.margin =unit(c(.5,.5,.5,.5),"cm"),
      plot.title = element_text(size = 11)
    )
  
  return (p)
}

##

layout <- rbind(c(1,2),c(3,4))


p<- doVPlot(res_human, "Covid vs. Healthy")

show(p)
```

```
## Warning: Removed 3516 rows containing missing values (geom_point).
```

# Filter for NAD genes

```
nad <- read.table("nad.genes.txt", sep='\t', header=TRUE)


# check which genes in results


nad_in_results <- res_human %>% as.data.frame() %>% tibble::rownames_to_column(var = "gene")  %>% pull(gene)

nad_not_in_results <- setdiff(nad$gene, nad_in_results)

nad_not_in_results
```

```
## [1] "NAPRT" "NADK2" "NOCT"
```

```
##
```

*Three NAD genes not present in the download dataset*

# Excel spreadsheets of NAD genes

```
library(writexl)

# excel files based on nad filter

res_human %>% as.data.frame() %>% rownames_to_column( var = "gene") %>% filter(gene %in% nad$gene)  %>% dplyr::select(-lfcSE,-stat) %>% arrange(padj) %>% write_xlsx(path ="human.cov.vs.healthy.xlsx")


res_human %>% as.data.frame() %>% rownames_to_column( var = "gene") %>% filter(gene %in% nad$gene)  %>% dplyr::select(-lfcSE,-stat) %>% arrange(padj) %>% filter(padj < 0.1)  %>%  kable() %>% kable_styling()
```

| gene | baseMean | log2FoldChange | pvalue | padj |
| --- | --- | --- | --- | --- |
| IDO1 | 1861.44349 | 4.600872 | 0.0000114 | 0.0006772 |
| ZC3HAV1 | 1106.50634 | 3.414592 | 0.0000283 | 0.0013274 |
| CD38 | 35.75037 | 3.497952 | 0.0028289 | 0.0403856 |
| PARP9 | 504.93939 | 2.757483 | 0.0037630 | 0.0465902 |
| NAMPT | 6470.90152 | 2.182501 | 0.0134003 | 0.0945876 |

# Session Info

```
sessionInfo()
```

```
## R version 3.5.2 (2018-12-20)
## Platform: x86_64-apple-darwin15.6.0 (64-bit)
## Running under: macOS Mojave 10.14.6
## 
## Matrix products: default
## BLAS: /Library/Frameworks/R.framework/Versions/3.5/Resources/lib/libRblas.0.dylib
## LAPACK: /Library/Frameworks/R.framework/Versions/3.5/Resources/lib/libRlapack.dylib
## 
## locale:
## [1] en_US.UTF-8/en_US.UTF-8/en_US.UTF-8/C/en_US.UTF-8/en_US.UTF-8
## 
## attached base packages:
##  [1] grid      parallel  stats4    stats     graphics  grDevices utils    
##  [8] datasets  methods   base     
## 
## other attached packages:
##  [1] writexl_1.1                 biomaRt_2.38.0             
##  [3] gridExtra_2.3               kableExtra_1.1.0           
##  [5] knitr_1.26                  tibble_2.1.3               
##  [7] ggrepel_0.8.1               ggplot2_3.2.1              
##  [9] DESeq2_1.22.2               SummarizedExperiment_1.12.0
## [11] DelayedArray_0.8.0          BiocParallel_1.16.6        
## [13] matrixStats_0.55.0          Biobase_2.42.0             
## [15] GenomicRanges_1.34.0        GenomeInfoDb_1.18.2        
## [17] IRanges_2.16.0              S4Vectors_0.20.1           
## [19] BiocGenerics_0.28.0         stringr_1.4.0              
## [21] dplyr_0.8.3                
## 
## loaded via a namespace (and not attached):
##  [1] bitops_1.0-6           bit64_0.9-7            progress_1.2.2        
##  [4] webshot_0.5.2          RColorBrewer_1.1-2     httr_1.4.1            
##  [7] tools_3.5.2            backports_1.1.5        R6_2.4.1              
## [10] rpart_4.1-15           Hmisc_4.3-0            DBI_1.0.0             
## [13] lazyeval_0.2.2         colorspace_1.4-1       nnet_7.3-12           
## [16] withr_2.1.2            prettyunits_1.0.2      tidyselect_0.2.5      
## [19] bit_1.1-14             compiler_3.5.2         rvest_0.3.4           
## [22] htmlTable_1.13.3       xml2_1.2.2             labeling_0.3          
## [25] scales_1.1.0           checkmate_1.9.4        readr_1.3.1           
## [28] genefilter_1.64.0      digest_0.6.23          foreign_0.8-72        
## [31] rmarkdown_1.16         XVector_0.22.0         base64enc_0.1-3       
## [34] pkgconfig_2.0.3        htmltools_0.4.0        highr_0.8             
## [37] htmlwidgets_1.5.1      rlang_0.4.2            rstudioapi_0.10       
## [40] RSQLite_2.1.2          farver_2.0.2           jsonlite_1.6          
## [43] acepack_1.4.1          RCurl_1.95-4.12        magrittr_1.5          
## [46] GenomeInfoDbData_1.2.0 Formula_1.2-3          Matrix_1.2-17         
## [49] Rcpp_1.0.3             munsell_0.5.0          lifecycle_0.1.0       
## [52] stringi_1.4.4          yaml_2.2.0             zlibbioc_1.28.0       
## [55] blob_1.2.0             crayon_1.3.4           lattice_0.20-38       
## [58] splines_3.5.2          annotate_1.60.1        hms_0.5.3             
## [61] locfit_1.5-9.1         zeallot_0.1.0          pillar_1.4.3          
## [64] geneplotter_1.60.0     XML_3.98-1.20          glue_1.3.1            
## [67] evaluate_0.14          latticeExtra_0.6-28    data.table_1.12.8     
## [70] vctrs_0.2.1            gtable_0.3.0           purrr_0.3.3           
## [73] assertthat_0.2.1       xfun_0.11              xtable_1.8-4          
## [76] survival_2.44-1.1      viridisLite_0.3.0      AnnotationDbi_1.44.0  
## [79] memoise_1.1.0          cluster_2.1.0
```
